# Organic Synthesis and Current Understanding of the Mechanisms of CFTR Modulator Drugs Ivacaftor, Tezacaftor, and Elexacaftor

**DOI:** 10.3390/molecules29040821

**Published:** 2024-02-10

**Authors:** Filipa C. Ferreira, Camilla D. Buarque, Miquéias Lopes-Pacheco

**Affiliations:** 1 Biosystems & Integrative Sciences Institute (BioISI), Faculty of Sciences, University of Lisbon, 1749-016 Lisbon, Portugal; 2 Department of Chemistry, Pontifical Catholic University of Rio de Janeiro (PUC-Rio), Rio de Janeiro 22435-900, RJ, Brazil

**Keywords:** CFTR corrector, CFTR potentiator, cystic fibrosis, drug development, mechanism of action, molecular structure, synthetic route, VX-445, VX-661, VX-770

## Abstract

The monogenic rare disease Cystic Fibrosis (CF) is caused by mutations in the gene encoding the CF transmembrane conductance (CFTR) protein, an anion channel expressed at the apical plasma membrane of epithelial cells. The discovery and subsequent development of CFTR modulators—small molecules acting on the basic molecular defect in CF—have revolutionized the standard of care for people with CF (PwCF), thus drastically improving their clinical features, prognosis, and quality of life. Currently, four of these drugs are approved for clinical use: potentiator ivacaftor (VX-770) alone or in combination with correctors lumacaftor, (VX-809), tezacaftor (VX-661), and elexacaftor (VX-445). Noteworthily, the triple combinatorial therapy composed of ivacaftor, tezacaftor, and elexacaftor constitutes the most effective modulator therapy nowadays for the majority of PwCF. In this review, we exploit the organic synthesis of ivacaftor, tezacaftor, and elexacaftor by providing a retrosynthetic drug analysis for these CFTR modulators. Furthermore, we describe the current understanding of the mechanisms of action (MoA’s) of these compounds by discussing several studies that report the key findings on the molecular mechanisms underlying their action on the CFTR protein.

## 1. Introduction

Mutations in the gene encoding the cystic fibrosis transmembrane conductance regulator (CFTR) are the molecular cause of the progressive autosomal recessive disorder Cystic Fibrosis (CF), which affects more than 100,000 individuals worldwide [1,2,3]. Despite being a multisystemic disease, CF primarily affects the respiratory and digestive systems, with the progressive loss of lung function as the major cause of morbidity and mortality in people with CF (PwCF) [3,4,5].

**Figure 1:**
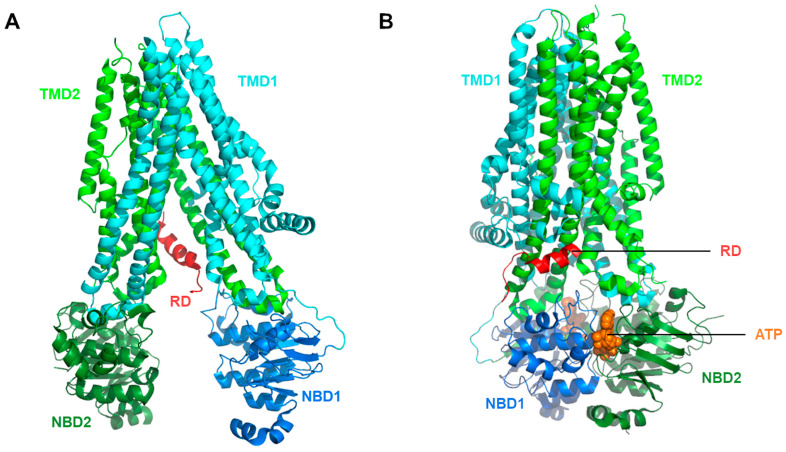
**Molecular structures of human CFTR conformations.** Ribbon diagram of (**A**) dephosphorylated ATP-free human CFTR conformation and (**B**) phosphorylated, ATP-bound human CFTR conformation. Adapted from PDB: 5UAK [9] and PDB: 6MSM [10], respectively, using PyMol Version 2.5.7.

The CFTR protein constitutes the only member from the ATP-binding cassette (ABC) transporter protein family that functions as an anion channel, transporting chloride and bicarbonate across the apical plasma membrane (PM) in epithelial cells [6,7,8]. The atomic structure of human CFTR was recently determined by cryogenic electron microscopy (cryo-EM) for the dephosphorylated ATP-free [9] and phosphorylated ATP-bound [10] conformations (Figure 1). As part of the ABC transporter sub-family C (ABCC), CFTR is also known as ABCC7, and its structure comprises two transmembrane domains (TMDs) and two cytosolic nucleotide-binding domains (NBDs), consisting of two homologous halves with TMD1 linked to NBD1 and TMD2 linked to NBD2 [5,11,12]. The CFTR protein further presents a unique regulatory domain (RD) linking TMD1/NBD1 to TMD2/NBD2 [7,11,13].

Mutations in the CFTR gene result in the absence (abrogation of protein biosynthesis or defective protein folding and trafficking) or dysfunction (defective channel gating/conductance) of the CFTR channel at the PM, leading to disrupted chloride/bicarbonate transport and compromised ion and fluid homeostasis at the epithelial lining surface [14,15,16]. Although over 2100 CFTR genetic variants have been reported so far, a three-base pair deletion (c.1521_1523delCTT) of the phenylalanine residue at position 508 (p.Phe508del, legacy name *F508del*) is the most prevalent pathogenic variant, occurring in approximately 80% of PwCF worldwide [1,3].

From the identification of the disease-causing gene [14,15,16], until recently, classical CF therapies have been focused on controlling and treating symptoms, thus acting downstream in the CF pathophysiologic cascade. However, since 2012 and over the last decade, novel therapies targeting the basic molecular defect(s) in CF have been approved for clinical use, termed CFTR modulators [3,17,18,19]. Among these drugs, potentiators are compounds that enhance CFTR gating by increasing channel open probability (Po), while correctors are small molecules that improve CFTR protein folding, thus restoring its trafficking to the PM. To date, one single molecule and three combinations of CFTR modulators have been approved for PwCF carrying specific CF-causing variants: potentiator ivacaftor (VX-770) as a single agent or in combinations with correctors lumacaftor (VX-809), tezacaftor (VX-661), and elexacaftor (VX-445). These drugs have provided life-changing perspectives for the majority of PwCF with impressive clinical benefits and improvements in quality of life. However, despite this enormous progress, PwCF undergoing CFTR modulator therapy still face several symptoms and complications, and not all PwCF are eligible for these treatments [20]. This indicates that there is still scope for additional and/or better combinations of CFTR modulators to further rescue mutant CFTR.

Aiming to efficiently obtain clinically relevant molecules such as CFTR modulators, more versatile and faster synthetic routes have been developed. Hughes made a noteworthy contribution to the Patent Review, extensively examining the synthetic routes of the clinically approved modulators ivacaftor, lumacaftor, tezacaftor, and elexacaftor [21]. Additionally, Flick and collaborators conducted a comprehensive review of the synthetic routes for tezacaftor [22]. In the scope of this review, we present a detailed retrosynthetic drug analysis for the three compounds, ivacaftor, tezacaftor, and elexacaftor, which compose a combinatorial “highly effective” modulator therapy (Trikafta^®^ in Boston, MA, USA and Kaftrio^®^ in Amsterdam, The Netherlands). We aim to uncover the nuanced interconnections among their constituent building blocks, placing particular emphasis on the most recent synthetic routes elucidated by Vertex Pharmaceuticals. Moreover, these modulator drugs rescue the mutant CFTR protein through diverse mechanisms of action (MoA’s), interacting with CFTR domains at different binding sites. Such knowledge provides valuable information not only to understand how these drugs work but also to develop more potent and effective therapies. Accordingly, we also discuss the findings on their corresponding MoA that have been reported to date.

## 2. Ivacaftor (VX-770)

**Figure 2:**
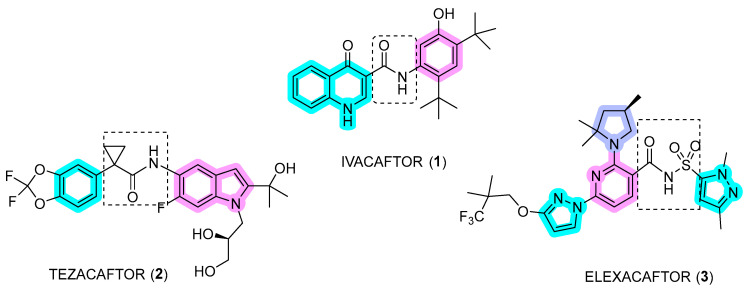
Chemical structures of ivacaftor (**1**), tezacaftor (**2**), and elexacaftor (**3**).

Ivacaftor possesses a molecular structure characterized by the presence of an *N*-(2,4-di-tert-butyl-5-hydroxyphenyl)-4-oxo-1,4-dihydro-quinoline-3-carboxamide moiety (Figure 2). The quinolone scaffold within its composition is a crucial pharmacophore, significantly influencing drug discovery. This scaffold holds prominence as one of the primary classes of nitrogen-containing heterocycles found in various biologically active compounds and blockbuster drugs, as highlighted in the literature [23,24]. The amide group serves as a crucial link between the “privileged building block” and the di-tert-butylphenol in ivacaftor’s structure. This linkage is of considerable importance in medicinal chemistry due to its multifaceted role. First, the amide group facilitates interactions with binding sites through hydrogen bonding, contributing to the compound’s pharmacological activity. Additionally, the amide bond’s metabolic instability towards enzymatic degradation is noteworthy, adding an element of controlled release and potentially impacting the compound’s overall pharmacokinetics. The high polarity of the amide group further influences the compound’s physicochemical properties and its interactions within biological systems, as discussed in the literature [25].

### 2.1. Synthetic Routes

**Scheme 1:**
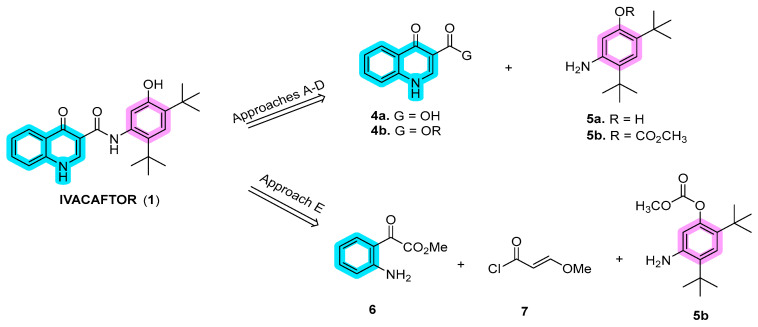
Disconnection approaches for the synthesis of ivacaftor (**1**).

In recent years, several synthetic routes have been explored for ivacaftor, primarily focusing on the synthesis of dihydroquinoline fragments **4a** or **4b** (Scheme 1). These fragments are subsequently connected to 5-amino-2,4-di-tert-butyl-phenol (**5a**) or its protected derivative **5b** through amide bond coupling, as outlined in approaches A to D. An alternative method involves the synthesis of the quinoline moiety in the final stages, commencing with methyl anthranilate, (*E*)-3-methoxyacryloyl chloride, and 5-amino-2,4-di-tert-butylphenyl methyl carbonate (**5b**) (approach E) [21].

**Scheme 2:**
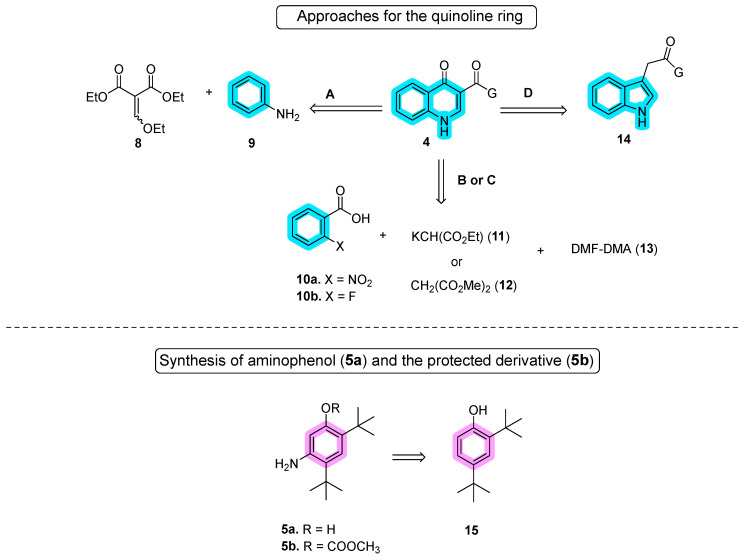
Different approaches for the synthesis of quinoline and aniline moieties.

In the first stage of synthesizing quinoline moiety **4** (approaches A–D), Vertex introduced the Gould–Jacobs reaction using diethyl ethoxymethylene malonate (**8**) and aniline (**9**), as documented in a pivotal work [26]. Vertex has adapted and modified this route over the years to meet the demands of large-scale production [27,28]. Approaches B and C share similarities and were independently developed by the Shanghai University of Engineering Science [29] and Laurus Pharma [30]. These results are outlined in Scheme 2. Yang’s approach started from *o*-nitrobenzoyl acid (**10a**), while Laurus Pharma initiated the synthesis with *o*-fluoro-benzoyl acid (**10b**). In approach D, Vasudevan and co-authors reported the synthesis of ivacaftor using the Witkop–Winterfeldt oxidation of indolyl group **14** to form the quinoline through ozone oxidation [31,32]. For amide bond coupling with quinoline, Vertex employed aniline **5a** in the initial route or 5-amino-2,4-di-tert-butylphenyl methyl carbonate (**5b**) in the second improved route, both derived from di-*tert*-butyl-phenol (**15**). In approach B, only aniline **5a** was utilized [33], whereas in approach E, **5b** was used instead. This intricate synthesis strategy showcases the diverse approaches taken by various researchers in the development of ivacaftor.

**Scheme 3:**
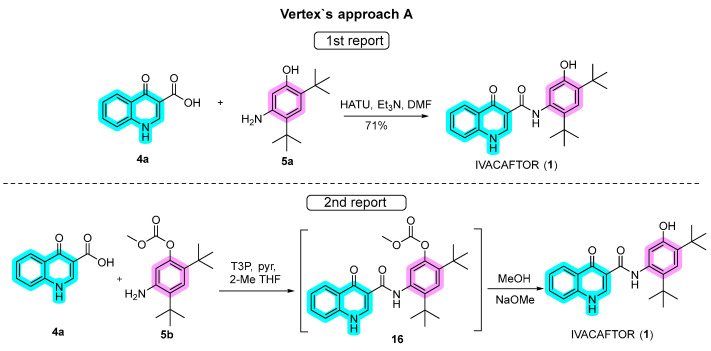
Approaches for the synthesis of ivacaftor used by Vertex.

Approach A, as employed by Vertex in the synthesis of ivacaftor (Scheme 3), is noteworthy for its efficiency. In the initial report of this approach, quinoline **4a** was synthesized through a three-step convergent route utilizing the Gould–Jacobs reaction, while aniline **5a** was obtained in four steps, as detailed below. The pivotal connection of these building blocks occurred through amide bonding coupling using 1-[bis(dimethylamino)methylene]-1*H*-1,2,3-triazolo[4,5-b]pyridinium 3-oxide hexafluorophosphate (HATU), in dimethylformamide (DMF). The reaction typically involves the activation of a carboxylic acid group to form an active intermediate, which then reacts with an amine to produce the amide bond with a high coupling efficiency in relatively mild reaction conditions. Subsequent purification by column chromatography resulted in ivacaftor with a notable 71% yield [34,35,36,37].

In a subsequent report, refinements to the synthetic route were implemented. Protected aniline **5b** replaced **5a**, and amide coupling was facilitated by propanephosphonic acid anhydride (T3P) with pyridine in 2-methyltetrahydrofuran (2-MeTHF), an alternative to HATU. While the reason for the switch to T3P as a coupling agent is not evident, it exhibits effectiveness under relatively mild conditions, reducing the risk of side reactions or unwanted byproducts. Additionally, it is compatible with a wide range of functional groups, rendering it suitable for use with a variety of substrates. Furthermore, T3P is a crystalline solid, which generally makes it easy to handle and store. Despite an additional protection step, intermediate **16** was conveniently hydrolyzed to ivacaftor in the same pot by the addition of MeOH and MeONa. A notable advantage of this modified approach was the elimination of chromatography steps, although specific yields for each step were not provided.

**Scheme 4:**
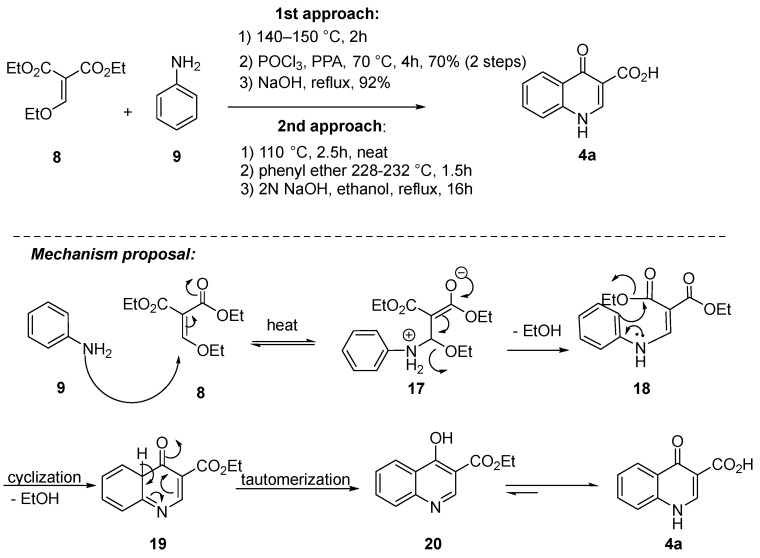
Gould–Jacobs reaction as a key step for the synthesis of quinoline **4a**.

Beyond the choice of amide coupling and/or the use of protected or deprotected aniline in the final step, notable advancements have been made in the synthesis of quinoline **4b** and aniline **5a** or its derivative **5b** in recent years (Scheme 4). For quinoline **4b**, the Gould–Jacobs reaction played a crucial role in generating quinolone **4a**. This involved a Claisen condensation between aniline **9** and diethyl ethoxymethylene malonate **8**, followed by Friedel−Crafts cycloacylation. The methodologies differed in some aspects. In the initial approach, diethyl ethoxymethylene malonate **8** reacted with aniline **9** under neat conditions for 2 h at 140–150 °C to yield enamine **18**. Subsequently, a POCl_3_/polyphosphoric acid (PPA) mixture was employed for the Friedel–Crafts cycloacylation through intermediates **19** and **20** [38], resulting in a 64% yield over the three steps. The mixture of POCl_3_/PPA is important to facilitate the cyclization process. POCl_3_ may potentially convert carboxylic acid, originating from the hydrolysis of the ester in the reaction medium, into acyl chloride. Simultaneously, dehydrating agent PPA aids in the removal of water molecules from the reaction mixture. In the second approach, enamine **18** was generated under neat conditions for 2.5 h at 110 °C, followed by the addition of diphenyl ether and heating to 228–232 °C for 1.5 h. Unfortunately, specific yields were not provided in this case [21,39,40].

**Scheme 5:**
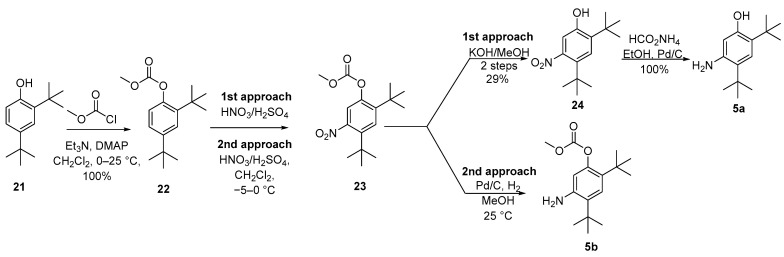
Synthesis of 5-amino-2,4-di-tert-butylphenol (**5a**) and 5-amino-2,4-di-*tert*-butylphenyl methyl carbonate (**5b**).

Since the coupling step with quinoline carboxylic acid **4a** may occur with aniline **5a** or **5b**, two reports were described for them. In the first report, a four-step route involved the protection of di-tert-butylphenol **21** to produce the methyl carbonate derivative **22** followed by nitration of **22** to afford **23** as a mixture of 8:1 of the desired 5-nitro regioisomer **23** and the undesired 6-nitro regioisomer. After hydrolysis with KOH in MeOH and purification by column chromatography, nitrophenol **24** was isolated in a 29% yield. Reduction of the nitro group employing transfer hydrogenation with ammonium formate led to **5a** in quantitative yield. In the second approach, nitration of carbonate **22** was performed in dichloromethane at −5 to 0 °C, and carbonate **23** was isolated by crystallization from hexane without the need for chromatography. The step of reduction of the nitro group occurred without deprotection of **23** by Pd catalyzed hydrogenation with a 2 bar hydrogen gas in MeOH, and product **5b** was purified by crystallization from MeOH/water (Scheme 5) [39,40]. The electron-withdrawing carbonate group may allow for nitration to occur primarily ortho/para to the tert-butyl groups since it minimizes the ortho/para-directing effect of the oxygen substituent. Data suggest that the second approach is the manufacturing of one European Public Assessment Report: Symkevi^®^ (26 July 2018) [41]. A summary of the synthetic methods and reaction conditions for the building blocks as well as ivacaftor are described in Supplementary Material (Supplementary Table S1).

### 2.2. Mechanisms of Action

Although the identification of ivacaftor by high-throughput (HT) screenings occurred in 2006, the first publications refer to 2009 as its discovery date. While ivacaftor was still in clinical development, Van Goor and colleagues [42] described the initial pharmacological properties of this potentiator in vitro, reporting its ability to partially restore CFTR activity. In this study, ivacaftor was found to increase the CFTR-mediated transepithelial current by increasing the CFTR Po, specifically after its activation by protein kinase A (PKA), in both cell lines and primary bronchial epithelial cells carrying variants p.PheF508del and/or p.Gly551Asp (legacy name *G551D*). The authors suggested that ivacaftor likely increases CFTR gating activity by directly binding to the protein [42], although at that time it was not yet elucidated whether it could instead act on an associated kinase or phosphatase.

The clinical approval of ivacaftor by both the US Food and Drug Administration (FDA) and the European Medicine Agency (EMA) came in 2012 initially for PwCF carrying at least one p.Gly551Asp [43] and extended in the following years for several gating/conductance variants [20,44,45]. In the same year of its approval, Eckford and collaborators [46] provided further insight into the MoA of ivacaftor by using a reconstitution system for purified CFTR protein. In this study, ivacaftor was found to bind directly to wild-type (WT) and mutant CFTR and stimulate the channel activity in an ATP-independent PKA phosphorylation-dependent process. This evidence suggested that ivacaftor binds to an allosteric site, distinct from the canonical, catalytic site, thus mediating CFTR channel potentiation by a nonconventional, ATP-independent mechanism of gating [46]. In a subsequent study, Jih and Hwang [47] performed electrophysiological recordings and used these results to propose a unified theory for the MoA of ivacaftor. Based on a CFTR gating model, in the presence of ATP, ATP-independent gating is an inherent component of the gating transitions observed for WT-CFTR. Additionally, this model includes a flexible coupling between ATP hydrolysis and gating events. These findings indicated that ivacaftor potentiates CFTR by promoting decoupling between gating cycles and ATP hydrolysis. The authors also suggested that the binding site of ivacaftor in CFTR is unlikely to be on the RD but instead may be located on the TMDs [47].

Two publications from independent research groups investigated whether ivacaftor was involved in the limited improvement of p.Phe508del-CFTR when co-administered with correctors, namely lumacaftor and tezacaftor [48,49]. Cholon and colleagues observed that, in primary cells, chronic exposure to ivacaftor promotes inhibition of lumacaftor-rescued p.Phe508del-CFTR by decreasing the stability of the corrected protein in a dose-dependent manner [48]. In parallel, Veit and collaborators also demonstrated that exposure to ivacaftor decreases the rescue efficacy of lumacaftor and tezacaftor in p.Phe508del-CFTR-expressing immortalized and primary cells [49]. Prolonged exposure (24 h) to ivacaftor reduced not only the folding efficiency but also the biochemical stability of p.Phe508del-CFTR. These findings were further confirmed by subsequent publications, demonstrating an inhibitory effect of ivacaftor on lumacaftor- or tezacaftor-rescued p.Phe508del-CFTR PM expression [50]. Similar effects were also observed in a later study in which long-term exposure to ivacaftor decreased elexacaftor-rescued p.Phe508del-CFTR [51]. In dynamics simulation and molecular docking studies, a putative binding site was proposed for ivacaftor in a region localized in the NBD1:NBD2 interface and the coupling helix of the intracellular loop (ICL) 1 [49].

In 2015, Yeh and co-authors [52] delved into the understanding of the modulation of CFTR channel gating by permeant ions, namely nitrate and ivacaftor. In this study, similarly to ivacaftor, nitrate increased the Po of the WT-CFTR channel, indicating that it has potentiator activity. Additive effects on CFTR gating were observed when both molecules were used together, suggesting that they work independently, through different binding sites to stimulate CFTR activity. Furthermore, this study proposed a putative binding site for ivacaftor on the TMDs of CFTR, at the interface between the membrane lipids and the protein channel [52]. These authors performed subsequent studies using electrophysiological recordings to further investigate ivacaftor and ABBV-974 (formerly GLPG1837) [53]. Interestingly, both potentiators were found to share a common mechanism to stimulate CFTR gating by competing for the same binding site, notwithstanding their variations in chemical structure, affinity, potency, and efficiency. This study also served as a basis to propose a four-state kinetic model based on a classic allosteric modulation model to explain the MoA and energetic coupling of potentiator binding and the opening of the CFTR channel [53]. In the following assessments, in silico molecular docking and electrophysiological assays were combined to provide further evidence of binding sites for ivacaftor and ABBV-974 [54]. Data from this study allowed for the identification of two potential binding sites located at the TMD1/2 interface, reinforcing the previous findings indicating that ivacaftor and ABBV-974 share the same binding site.

Another putative binding site for ivacaftor was proposed in the study of Byrnes and colleagues [55] using hydrogen/deuterium exchange (HDX) mass spectrometry to characterize CFTR conformational dynamics and its binding interactions with ligands. Using this approach, ivacaftor was suggested to bind to a region of amino acids in ICL4 of the “ball and socket” joint at the TMD2:NBD1 interface—a region close to where the p.Phe508 residue is present. This implies that HDX protection by ivacaftor at ICL4 may extend to the region of this residue as well. Using another approach, Csanády and Töröcsik [56] explored the solubility profile and potency of ivacaftor by stimulating WT and mutant CFTR channels in cell-free membrane patches. It was found that the aqueous solubility of ivacaftor is two orders of magnitude lesser than previously suggested [57]. Furthermore, CFTR stimulation by ivacaftor in cell-free patches was shown to be fully reversible, contrary to what was previously reported [47,52,53,58,59,60]. Based on these observations, the authors mentioned that up to that point, ivacaftor effects were assessed only at high supersaturated concentrations. This study also proposed a kinetic model for the MoA of ivacaftor in CFTR potentiation consisting of two independent binding sites with identical affinity, which thus requires that two molecules bind to the channel to potentiate CFTR [56].

**Figure 3:**
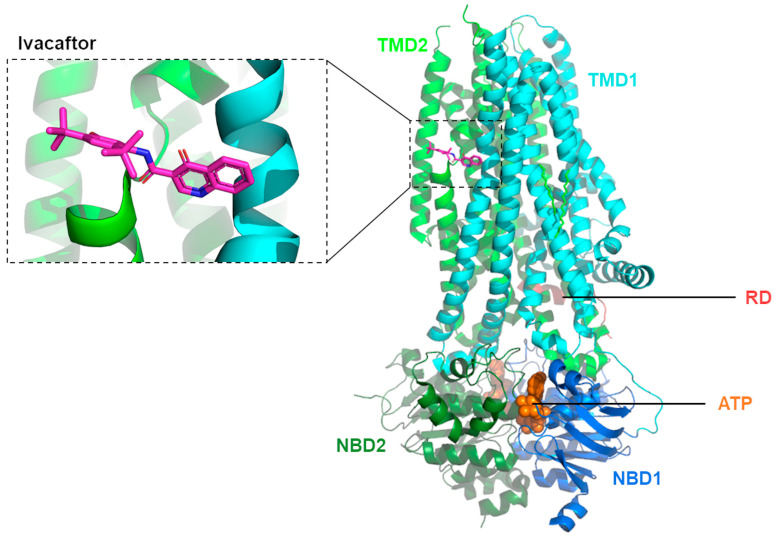
**Molecular structure of phosphorylated, ATP-bound human CFTR in complex with ivacaftor.** Ribbon diagram adapted from PDB: 6O2P [61] using PyMol Version 2.5.7.

Using cryo-EM, Liu and collaborators [61] were able to determine the structure of CFTR in complex with ivacaftor (PDB: 6O2P; Figure 3) and separately with ABBV-974 (PDB: 6O1V), allowing for more direct evidence of these potentiators’ binding site. These results validated previous reports that suggested a common binding site for both ivacaftor and ABBV-974 located in a region inside the lipid bilayer, within the protein–lipid interface of TMD1/2. This hotspot coincides with a hinge region involved in the gating of the channel. Such evidence allowed the authors to suggest that the open configuration of the CFTR channel is stabilized (instead of the closed) when a drug is present in that binding pocket [61]. A subsequent study by Righetti and collaborators [62] used this recently determined structure of human CFTR in complex with ivacaftor [61] to investigate the binding of potentiator to p.Phe508del-CFTR by combining molecular docking, pharmacophore mapping, and quantitative structure–activity relationship (SAR) analysis. By exploiting the most relevant amino acids involved in the ivacaftor binding site, the authors found key residues such as p.Phe931 and p.Arg933 that act via Van der Waals interactions, *H*-bonds, cation-π contacts and involve additional polar interactions in other residues to stabilize the binding of potentiators like ivacaftor [62].

Observing such contradictory results regarding the MoA of CFTR potentiation by ivacaftor, Laselva and collaborators [63] decided to re-evaluate these putative binding sites in the natural context of this channel protein in the lipid bilayer by using photoactivatable ivacaftor probe analogs. With the evidence obtained in this study, the authors proposed a model with two specific binding sites for ivacaftor: one in ICL4 at the NBD1:TMD2 interface and the other in the region of TMD1/2 and membrane lipid interface, as identified by cryo-EM [61]. Whether ivacaftor stabilizes CFTR open channel configuration by binding to ICL4 independently or in conjunction with its binding in the other region remains to be further elucidated [63].

Recently, Levring and collaborators [64] explored SAR in human WT-CFTR at a single-molecule resolution, combining ensemble ATPase activity measurements, single-molecule fluorescence resonance energy transfer (FRET), imaging, electrophysiology, and kinetic simulations. Using this integrative approach, the authors showed the occurrence of dimerization of the two NBDs before the channel opening, revealing an allosteric gating mechanism involving the channel pore and the catalytical binding site. Furthermore, it was observed that potentiators ivacaftor and ABBV-974 act on CFTR and enhance channel activity by increasing pore opening while the NBDs are dimerized, thus influencing the coupling efficiency between ion permeation and NBD dimerization [64]. More recent insights into the mechanisms underlying the action of ivacaftor were described by Ersoy and colleagues [65] who investigated allosteric communications in the CFTR protein by using computational analysis. The authors observed that the binding site for ivacaftor comprises some residues that are main allosteric sources, suggesting a role for this compound as an allosteric modulator. Furthermore, it was found that ivacaftor’s binding site shares similarities with the ATP binding site as both send information to an almost identical set of residues, suggesting that ivacaftor indirectly increases the Po by replicating the combined allosteric signaling triggered by the binding of ATP and by gating residues [65].

It should be noted that, despite the therapeutic accomplishments of ivacaftor, it only attains a partial restoration of the CFTR gating activity [42,47,66]. Accordingly, novel potentiators have been investigated, and their combination with complementary mechanisms (i.e., co-potentiators) has emerged as a strategy to further enhance CFTR gating activity [66,67,68,69,70,71].

## 3. Lumacaftor (VX-809), Tezacaftor (VX-661) and Double Combinations with Ivacaftor

In contrast to ivacaftor, tezacaftor features a sophisticated molecular structure comprising (*S*)-1-(2,2-difluorobenzo[d][1,3]dioxol-5-yl)-*N*-(1-(2,3-dihydroxypropyl)-6-fluoro-2-(2-hydroxypropan-2-yl)-1*H*-indol-5-yl)cyclopropane-1-carboxamide moiety (Figure 2). The pivotal connector in this arrangement is a cyclopropylamide, seamlessly linking the difluorobenzodioxole scaffold to the substituted indole. The cyclopropyl ring, recognized as a versatile entity, holds prominence in the realm of preclinical/clinical drug molecules. Its versatile utility extends to serving as an isosteric substitute for an alkene, strategically enhancing potency, boosting metabolic stability, mitigating off-target effects, improving brain permeability, reducing plasma clearance, and modifying drug pKa to diminish *P*-glycoprotein efflux [72]. Indole, a privileged scaffold and nitrogen-containing heterocycle, embellishes tezacaftor’s composition with a diverse array of pharmacological activities attributed to various MoAs [73]. The stereochemistry of the diol group linked to the indole, especially at the tertiary alcohol, assumes significance in its interaction with the binding sites of the CFTR. This stereochemical aspect contributes intricately to the compound’s ability to engage with specific molecular targets, adding a layer of precision to its pharmacological profile.

### 3.1. Synthetic Routes

**Scheme 6:**
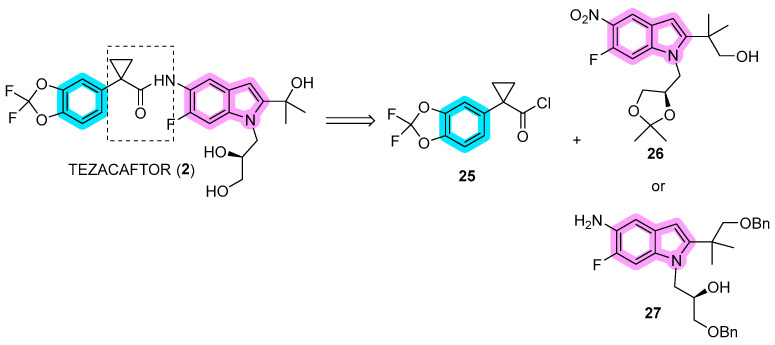
Different synthetic routes for tezacaftor employing building blocks **25** plus **26** or **27**.

Vertex outlined two synthetic routes for tezacaftor spanning two generations, distinguished by the utilization of building blocks **26** in the first generation and **27** in the second one (Scheme 6).

**Scheme 7:**
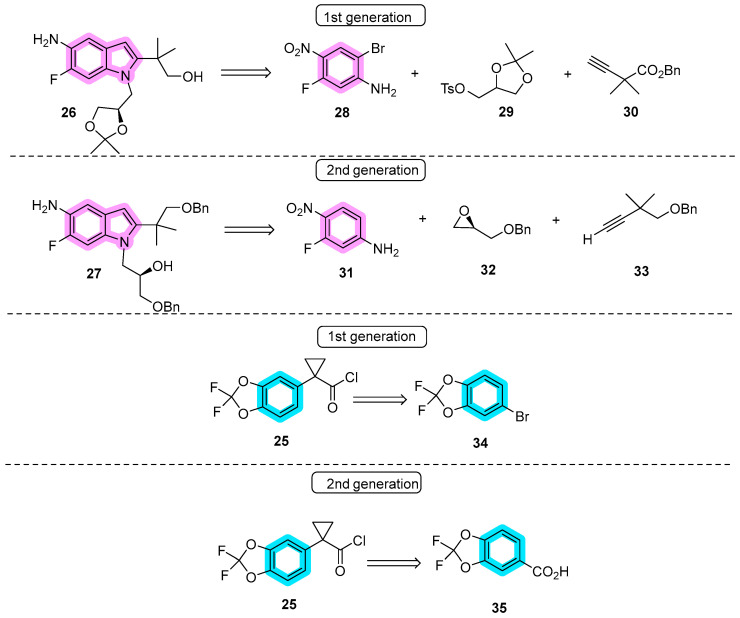
Building blocks involved in the synthesis of tezacaftor.

Alkyne **30** played a pivotal role in the preparation of building block **26** during this first-generation synthesis. In contrast, the second-generation pathway adopted a more sophisticated starting material, alkyne **33**, which reduced the route by two steps. Building block **25** containing the difluorodioxole moiety was synthesized from aryl bromide **34**, yielding 1.6% efficiency across the final four steps in the first generation. In the second generation, enhanced efficiency and higher yields were achieved by starting from carboxylic acids **35** (Scheme 7).

**Scheme 8:**
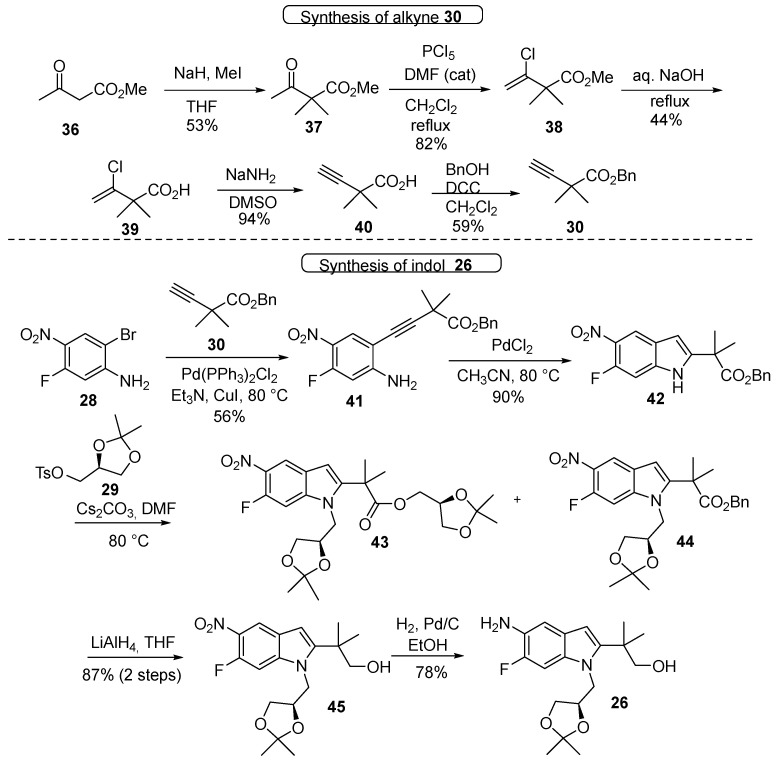
First-generation synthesis of alkyne fragment **30** and indol **26**.

The first-generation synthetic route to tezacaftor followed a convergent approach, requiring 17 steps and the longest linear sequence of 12 steps [74,75,76,77]. For the synthesis of alkyne **30**, methyl acetoacetate **36** underwent double methylation with MeI in THF, resulting in **37** with a 53% yield. Transformation to chloride **38** was achieved using PCl_5_ in dichloromethane with catalytic DMF, yielding 82% of the product. Hydrolysis of the ester produced carboxylic acid **39** in a 44% yield. The conversion to alkyne **40** was conducted using sodium amide in DMSO, yielding 94%. This was followed by the formation of alkyne **30** through DCC mediation in dichloromethane. This alkyne was used for Sonogashira cross-coupling with aryl bromide **28** in triethylamine as the solvent to furnish alkyne **41** in a 56% yield. Indole **42** was obtained in a 90% yield by the Larock reaction catalyzed by PdCl_2_ in acetonitrile at 80 °C. The alkylation of indole nitrogen with tosylate **29** in the presence of DMF with cesium carbonate at 80 °C led to a mixture of **43** and **44** that was reduced with LiAlH4 in THF to produce alcohol **45** in an 87% yield over two steps. Hydrogenation of the nitro group in EtOH afforded aniline **26** in a 79% yield (Scheme 8) [74,75,76].

**Scheme 9:**
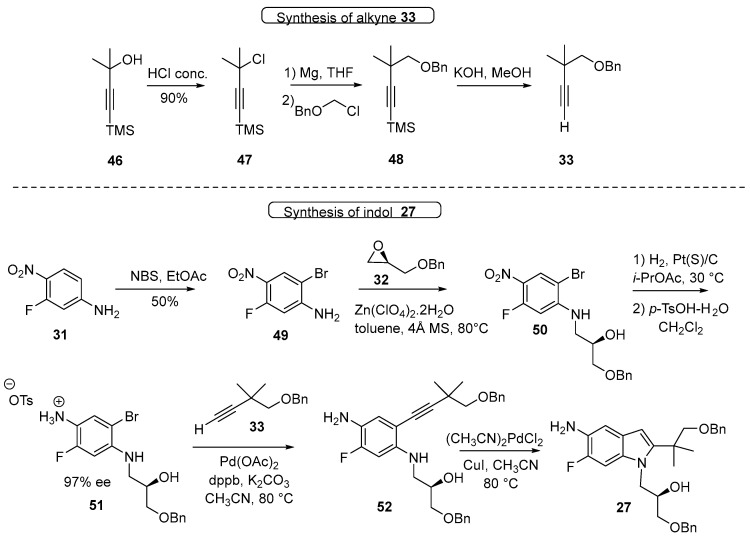
Second-generation synthesis of alkyne fragment **33** and indol **27**.

The second-generation synthetic route to tezacaftor takes a total of 15 steps, featuring the longest linear sequence of seven steps [78,79]. In the synthesis of alkyne **33**, the conversion of alcohol **46** to tertiary chloride **47** achieved a high yield of 90% using concentrated HCl. Subsequently, the formation of the Grignard complex, followed by alkylation with chloromethyl benzyl ether, yielded ether **48**. The TMS group was then cleaved with KOH in MeOH, resulting in the formation of fragment **33**, although specific yield information was not provided. The synthesis of alkyne **33** played a pivotal role in the copper-free Sonogashira step, transforming compound **51** (in its free base form) to **52**. To prepare building block **51**, the synthetic route initiated with the bromination of 3-fluoro-4-nitroaniline (**31**) using *N*-bromosuccinimide (NBS) in EtOAc to yield **49** in a 50% yield after crystallization. At this stage, the alkylation of **49** with the chiral side chain occurred through the ring opening of (*R*)-benzyl glycidyl ether (**32**), catalyzed by zinc perchlorate dihydrate and molecular sieves in toluene at 80 °C. Compound **52** was not isolated, but instead there was a solvent switched to *i*-PrOAc for the subsequent step. The cyclization of crude **52** was catalyzed by (MeCN)_2_PdCl_2_ (15%) in MeCN at 80 °C, resulting in the formation of indole **27** in an overall yield of 70% over the two steps (Scheme 9).

As described before, the synthesis route leading to difluorodioxole fragment **25** in the first-generation route from aryl bromide **34** yielded 1.6% efficiency across the final four steps in the first generation (refer to Supplementary Material: Supplementary Table S2).

The second-generation route began with the usage of commercially available carboxylic acid **35**. This starting material was efficiently reduced to alcohol **53** using sodium bis(2-methoxyethoxy)aluminum hydride (Vitride) in toluene, providing yields of 86–92%, obviating the need for purification. Crude product **53** underwent further transformation with thionyl chloride (SOCl_2_) and catalytic 4-(dimethylamino)pyridine (DMAP) (1%) in methyl *tert*-butyl ether (MTBE), yielding **54** with remarkable yields ranging from 82 to 100%, again without requiring purification. The subsequent conversion of **54** to nitrile **55** was achieved using NaCN in DMSO by an S_N_2 reaction, resulting in excellent yields of 95–100%. For the introduction of the cyclopropane motif, 1,2-bromochloroethane was employed under phase-transfer conditions, utilizing tetraoctylammonium bromide and 50% KOH at 70 °C, yielding **56** in the range of 88–100%. Nitrile **56** underwent hydrolysis employing 6 N NaOH in EtOH to yield carboxylic acid **57** in a satisfactory yield of 69% and after purification through crystallization. The treatment of **57** with SOCl_2_ in DMF led to **25** in six steps from **35**. The yield of the last step was not provided.

**Scheme 10:**
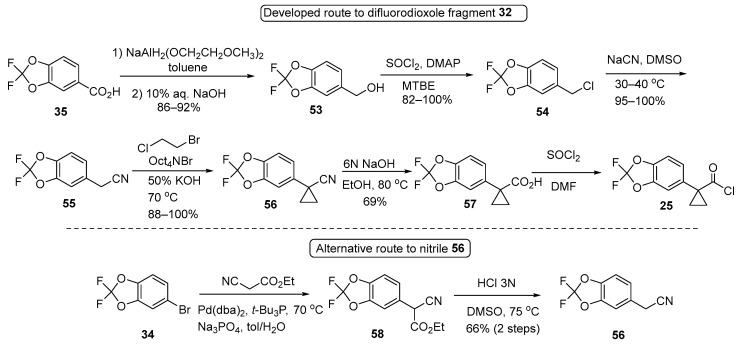
Synthesis of difluorodioxazole building block **25**.

As an alternative approach to accessing nitrile **55** following the procedure described by Jung and collaborators [80], palladium-catalyzed cross-coupling of aryl bromide **34** with ethyl cyanoacetate was employed, furnishing cyano ester **58**. Subsequent ester hydrolysis and decarboxylation using 3N HCl at 75 °C resulted in the production of nitrile **56** in a yield of 66% after purification through vacuum distillation (Scheme 10).

**Scheme 11:**
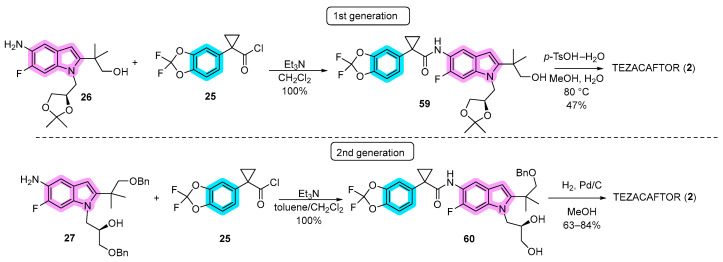
First- and second-generation routes for the synthesis of tezacaftor (**2**).

The conclusive steps leading to the synthesis of tezacaftor are elucidated in Scheme 11. In the first generation, the coupling of aniline **26** with acid chloride **25** in dichloromethane provided amide **59** in a quantitative yield. Deprotection using *p*-TsOH-H_2_O in MeOH at 80 °C furnished tezacaftor in a 47% yield after purification by flash chromatography. In the second generation, the amide bond formation between compound **27** and acid chloride **25** was facilitated by triethylamine in toluene/dichloromethane; the process yielded tezacaftor precursor **60**. Subsequent hydrogenation utilizing Pd/C in MeOH effectively cleaved benzyl groups, yielding tezacaftor in yields ranging from 63% to 84% over two steps. The final product was obtained after crystallization from a mixture of 2-PrOH/heptane. A summary of the synthetic methods and reaction conditions for the building blocks as well as tezacaftor are described in Supplementary Material (Supplementary Table S2).

### 3.2. Mechanisms of Action

The screening of compound libraries by cell-based HT assays led to the identification of several chemical compounds that act as CFTR correctors [20,81,82,83]. One of the active compounds identified in these assays—VRT-768—was extensively investigated to improve the chemical properties of this scaffold and thus obtain improved analogs. This work eventually resulted in the identification of a first-generation corrector–lumacaftor whose initial pharmacological properties in vitro were described in 2011 by Van Goor and collaborators [84]. In terms of the MoA of lumacaftor, the results suggested that this corrector should interact directly with either CFTR or CFTR-associated proteins, specifically targeting the processing defect of p.Phe508del-CFTR [84]. Subsequent studies by Sinha and co-authors confirmed the direct binding of this compound to the CFTR protein [85]. Using click chemistry approaches, these provided in vitro evidence of direct binding of generated lumacaftor derivatives to WT- and p.Phe508del-CFTR [85].

Several studies further investigated lumacaftor while the corrector was still undergoing clinical development. He and colleagues [86] found that similarly to other small-molecule correctors, incubation of cells with lumacaftor at a lower temperature (27 °C) resulted in a significant increase in p.Phe508del-CFTR processing rescue, indicating that this corrector alone is unable to restore the thermodynamic stability of the mutant protein. This study also suggested the NBD1:ICL4 interface as one possible location for the binding of lumacaftor [86]. In parallel, Okiyoneda and co-authors [87] performed in silico, in vitro, and in vivo studies, and proposed a classification system for CFTR correctors (or pharmacological chaperones) based on their mechanistic targets: the NBD1:TMD1 or NBD1:TMD2 interfaces (type I), NBD2 (type II) or the p.Phe508del-NBD1 energetic defect (type III). As previously assessed [86], the functional stability of a temperature-rescued mutant CFTR was investigated and the results provided further evidence that lumacaftor can interact directly with p.Phe508del-CFTR at a single-molecule level [87]. Even though lumacaftor prevented functional inactivation when increasing the temperature from 24 °C to 36 °C, prolonged incubation at the physiological temperature (37 °C) reduced the rescue effect of lumacaftor, which was suggested to occur due to the defective stabilization of NBD1:TMD1 and NBD1:TMD2 interfaces [87]. Overall, this evidence further indicated the NBD1:ICL4 interface as the possible primary target of lumacaftor. In a third study, Farinha and collaborators [88] explored the MoA of lumacaftor by assessing its additive/synergistic effects when combined with p.PheF508del-CFTR genetic revertants (p.Val510Asp, p.GlyG550Glu, p.Arg555Lys, p.Arg1070Trp, and 4RK), other investigational correctors (Corr-4a and VRT-325) and low-temperature incubation. Although lumacaftor exerted variable effects on genetic revertants, it was found that the rescue of p.Phe508del-CFTR by this compound was additive to p.GlyG550Glu, p.Arg555Lys, and 4RK as well as the two other correctors and low-temperature incubation [88]. This evidence strongly suggested that lumacaftor acts on the mutant protein by a distinct MoA compared to those other rescuing strategies. Consistent with concurrent findings [50,86,87], modeling and docking data supported the NBD1:ICL4 interface as the putative binding site for lumacaftor [88]. Similar findings were observed in subsequent studies investigating the putative binding site of tezacaftor [50,89].

However, some uncertainty persisted whether lumacaftor docks TMD2. Indeed, in that same year, results from two publications pointed instead to lumacaftor binding to TMD1 [90,91]. Loo and Clarke [90] investigated whether lumacaftor directly interacts with the TMDs of the CFTR protein using truncation mutants and isolated CFTR domains. This analysis evidenced TMD1, TMD2, and NBD1 as essential domains for CFTR maturation promoted by lumacaftor. The corrector was also able to specifically stabilize TMD1—but not the other domains—increasing its half-life by five-fold, which suggests that this domain should contain a binding site for lumacaftor [90]. Simultaneously, Ren and colleagues [91] further explored the MoA of lumacaftor using other in vitro assays and found that this corrector exerts its action at an early stage of CFTR biogenesis, modulating and increasing the stability of the protein conformation of TMD1 to partially correct and prevent folding defects in p.Phe508del-CFTR. Considering the evidence from He and others [86], the authors suggested that the conformation alteration of the specific region of TMD1 by lumacaftor also suppresses the defects in ICL4 by an allosteric mechanism, hence the corrector does not directly act on the NBD1:ICL4 interface [91].

To better understand the MoA of CFTR correctors, Eckford and co-authors [92] studied lumacaftor and the structurally related corrector C18 (formerly VRT-534 [81]) in purified full-length WT- and p.Phe508del-CFTR protein in vitro. Based on the hypothesis that some correctors might have multiple effects on the mutant protein, the authors investigated whether these compounds exert other effects in addition to the rescue of p.Phe508del-CFTR processing. Results indicated that both lumacaftor and C18 have a secondary acute effect, directly interacting post-translationally with the full-length p.Phe508del-CFTR after its trafficking rescue to the PM, thus enhancing or stabilizing the mutant protein channel, although the impact on channel activity is not even closely comparable to the potentiation effect of ivacaftor [92]. Nevertheless, as previously reported [48,49], the rescue of p.Phe508del-CFTR by lumacaftor is reduced by chronic ivacaftor co-treatment as the last decreased stability of lumacaftor-rescued p.Phe508del-CFTR. Regarding C18, various studies demonstrated that its combination with Corr-4a increases the CFTR correction not only for p.Phe508del but also for several misfolded CFTR variants [86,87,88,93,94], suggesting that the combination of lumacaftor with another corrector sharing the MoA of Corr-4a may elicit further rescue of the CFTR protein.

The identification of the binding site in CFTR for lumacaftor remained a challenge. Two new publications [95,96] presented further evidence for each of the TMDs. Loo and Clarke previously reported stabilization of TMD1 by lumacaftor [90] and the new study [95] investigated the MoA of this corrector using CFTR truncation mutants. Following Ren and co-authors [91], the data indicated that lumacaftor promotes p.Phe508del-CFTR maturation and stabilization by a mechanism involving domain interactions between ICL1:NBD1, suggesting that lumacaftor directly binds to CFTR in TMD1 [95]. In contrast, Hudson and colleagues [96] used nuclear magnetic resonance spectroscopy and demonstrated that lumacaftor directly binds to WT- and p.Phe508del-NBD1 but acts on the TMD2. These results suggest that this direct binding promotes conformational changes in NBD1 and is allosterically coupled with the NBD1:ICL4 interface, justifying the exerted effects on this interface without requiring its direct binding, as reported in other previous studies [86,87].

The clinical approval of the combination of lumacaftor and ivacaftor occurred in 2015; however, this treatment only produced modest clinical benefits for p.Phe508del homozygous individuals [97]. Accordingly, further efforts were employed to identify more effective CFTR correctors. Based on the chemical structure of lumacaftor, a second-generation CFTR corrector—tezacaftor—was developed. As tezacaftor is chemically derived from lumacaftor, these correctors are postulated to share a common MoA in rescuing CFTR. Similarly to the precursor analog, the double combination of tezacaftor with prolonged treatment with ivacaftor impaired the rescue of p.Phe508del-CFTR [49,50,89].

Molinski and collaborators [98] explored the global landscape of known CFTR variants and searched for trends or patterns using a structural pharmacogenomics approach to comprehensively map these variants in the CFTR protein structure. Disease-causing variant clusters were found in specific regions of the CFTR structure, namely at NBD1:NBD2 and NBD1:ICL4 interfaces. These in vitro data were then combined with molecular docking to predict corrector-sensitive CFTR residues responsible for the putative binding site for type I correctors such as lumacaftor, tezacaftor, and C18. The findings confirmed that all three correctors stabilize TMD1, evidencing that they share a similar MoA, and suggesting that the most plausible binding pocket involves several residues from TMD1 (ICL1 and *C*-terminal) but also from other different domains (the *N*-terminal of NBD1, ICL4 in TMD2) [98]. Baatallah and co-authors [99] further explored the putative binding sites for lumacaftor and tezacaftor in both NBD1 and TMD1. Combining docking and molecular dynamics simulations with biochemical assays, two potential binding sites for lumacaftor/tezacaftor were observed: the first in a TMD1 groove with lumacaftor inducing a global folding stabilization, and the second within NBD1 with lumacaftor stabilizing the NBD1:ICL4 interface. The authors further suggested and described allosteric coupling linking the aforementioned first binding site to NBD1 in the region of the p.Phe508del variant [99].

Krainer and colleagues [100] investigated the rescue of the p.Val232Asp variant (legacy name *V232D*) by lumacaftor in a helical hairpin construct derived from full-length CFTR and containing CFTR’s transmembrane Helices 3 and 4. Using the single-molecule FRET assay to study misfolding, the p.Val232Asp hairpin resulted in an equilibrium shift towards an open conformation. Importantly, this aberrant opening and protein misfolding is reversed by incubation with lumacaftor, restoring a compact hairpin state characteristic of the WT hairpin and promoting proper folding. The authors thus suggested that rather than indirect effects, the rescue ability of this corrector could be due to a direct energetic effect allowed because the stabilization of the native CFTR conformation is preferred [100]. In a subsequent study [101], these authors employed the same hairpin construct and FRET approach to study lumacaftor effects on the loop p.Glu217Gly variant (legacy name *E217G*). Similar to the results in the p.Val232Asp variant, lumacaftor produced a dynamic helix stabilization effect on WT-CFTR and the p.Glu217Gly variant. These results suggest that lumacaftor has a wide mode of action probably due to its membrane-destabilizing capabilities through which the corrector effectively promotes CFTR folding stability and rescues misfolding and maturation of several different CFTR variants [101]. In another study, Ensinck and collaborators [102] investigated the effects of lumacaftor with and without ivacaftor on four rare CFTR variants, namely p.Glu60Lys, p.Gly85Glu, p.Glu92Lys, and p.Ala455Glu (legacy names *E60K*, *G85E*, *E92K*, and *A455E*, respectively). A workflow of complementary phenotypic, flow cytometry, and functional assays was developed to assess the maturation, PM trafficking, and function of these CFTR variants. Results demonstrated that lumacaftor was able to significantly rescue p.Glu60Lys- and p.Glu92Lys-CFTR function by promoting correct protein folding and PM trafficking. It was also suggested that variants p.Glu60Lys and p.Glu92Lys could likewise benefit from the clinically approved combination treatment of lumacaftor plus ivacaftor and by extrapolation of tezacaftor plus ivacaftor [102].

Biochemical approaches were employed by Amico and colleagues [103] to identify the CFTR domains involved in WT- and p.Phe508del-CFTR rescue by correctors and to explore the MoA of different correctors, namely lumacaftor, tezacaftor, VX-325, and Corr-4a. Whereas treatment with each of the four correctors did not produce an effect on either the expression or the maturation of WT-CFTR, it significantly increased the functional expression and maturation on full-length p.Phe08del-CFTR. Furthermore, the authors generated constructs of parts of p.Phe508del-CFTR, namely the *N*-half (residues 1–633) and the *C*-half (residues 837–1480), and found variable effects on the action of correctors selective for specific regions [103]. For instance, three correctors (lumacaftor, tezacaftor, and VX-325) increased the expression and maturation specifically of the WT- and p.Phe508del-CFTR *N*-halves, while Corr-4a selectively affected the expression and maturation of the p.Phe508del-CFTR *C*-half and the NBD2 domain [103]. Using another approach, Uliyakina and colleagues [104] assessed the effects of removing two unique conformationally dynamic regions from p.Phe508del-CFTR—the regulatory extension and the regulatory insertion—and the consequent impact on the rescue of this variant. The results demonstrated that p.Phe508del-CFTR processing without the regulatory insertion and without both regulatory regions was rescued by lumacaftor to WT-CFTR levels, although functional rescue required the removal of the two regions [104]. These findings suggest that the MoA of lumacaftor might also involve binding to the regulatory insertion.

**Figure 4:**
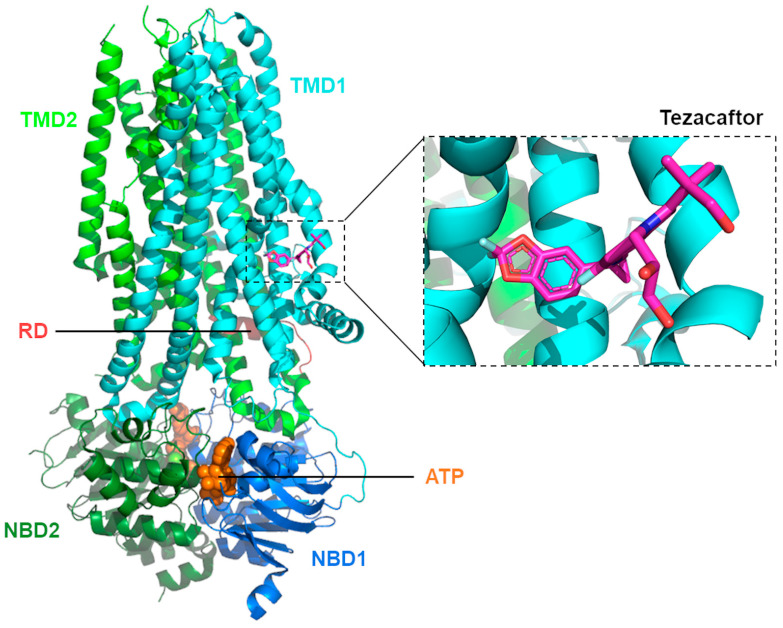
**Molecular structure of phosphorylated, ATP-bound human CFTR in complex with tezacaftor.** Ribbon diagram adapted from PDB: 7SV7 [106] using PyMol Version 2.5.7.

Further evidence on the mechanistic folding of individual domains (TMD1 and NBD1) was developed by Kleizen and co-authors [105], who also investigated the effects of lumacaftor on these models. Using biosynthetic radiolabeling and pulse-chase assays combined with protease susceptibility experiments, they found that the folding of hydrophobic and soluble helices from the TMD1 occurs co-translationally. It was also observed that lumacaftor promotes the assembly, packing, and stabilization of TMD1, suggesting that its action occurs early on during CFTR biosynthesis and the binding site for this corrector is close to this domain region [105]. More recently, the cryo-EM structures of human CFTR in complex with approved type I CFTR correctors lumacaftor (PDB: 7SVR, dephosphorylated, ATP-free CFTR; PDB: 7SVD, phosphorylated, ATP-bound CFTR) or tezacaftor (PDB: 7SV7, phosphorylated, ATP-bound CFTR; Figure 4) were determined by Fiedorczuk and Chen [106]. These findings further elucidated the MoA of these correctors, with both compounds binding directly to the same hydrophobic internal pocket in TMD1. Such evidence supports an MoA in which type I correctors such as lumacaftor and tezacaftor stabilize TMD1 early on CFTR biogenesis, preventing targeting for premature degradation, and thus promoting mutant CFTR rescue [106].

## 4. Elexacaftor (VX-445) and Triple Combination with Tezacaftor and Ivacaftor

Elexacaftor is defined by its molecular structure as (*R*)-*N*-((1,3-dimethyl-1*H*-pyrazol-5-yl)sulfonyl)-4-(3-(3,3,3-trifluoro-2,2-dimethylpropoxy)-1*H*-pyrazol-1-yl)-2-(2,2,4-trimethylpyrrolidin-1-yl)benzamide (Figure 2). A noteworthy feature of this composition is the presence of a linker sulfonamide—a structural motif with a rich history dating back to the development of sulfa drugs [107,108]. The sulfonamide linker, well-established in synthetic drugs, exemplifies its significance in elexacaftor’s design. Its historical connections in medicinal chemistry emphasize its lasting contribution to the evolution of therapeutic agents. Further contributing to elexacaftor’s pharmacological potential is the inclusion of the nitrogen-containing heterocycle pyrazole, a known pharmacophoric group in bioactive compounds [109]. The versatility of the pyrazole moiety, as evidenced by its presence in compounds like VRT-532 [4,81], adds a dimension of bioactivity to elexacaftor’s profile.

### 4.1. Synthetic Routes

**Scheme 12:**
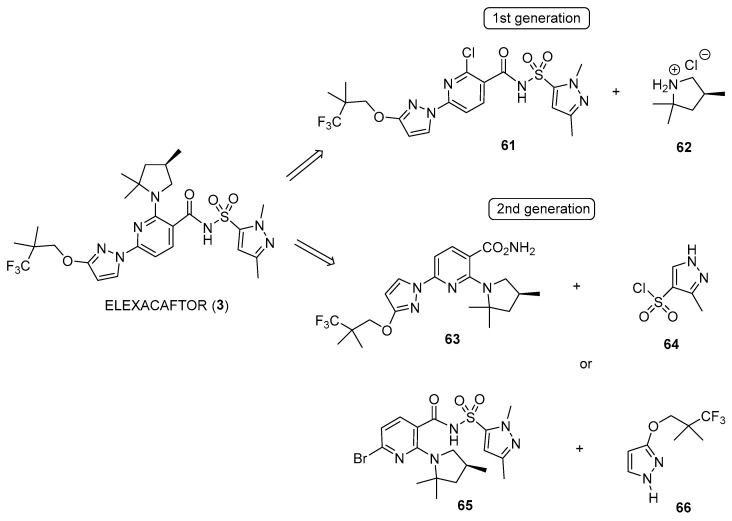
First and second generation for the synthesis of elexacaftor (**3**).

Similar to the synthesis of tezacaftor, Vertex described two generations of synthetic routes for elexacaftor. In the first generation, the connection involved building blocks **61** and **62**, while in the second generation, two alternative interconnections were explored. Specifically, the options included the linkage of building blocks **63** and **64** or the connection between **65** and **66** (Scheme 12).

First-generation synthesis entailed the union of pyrazoles **66** and **68** with 2,6-dichloropyridine-3-carboxylic acid **67** for the assembly of building block **61**. In contrast, the second-generation methodology diverged by substituting **67** with bromo-6-fluoronicotinic acid **69**. This alternative compound was then coupled either with pyrazole **66** and pyrrolidine **62** or with sulfonamide **64** and pyrrolidine **62** in the second-generation approach.

**Scheme 13:**
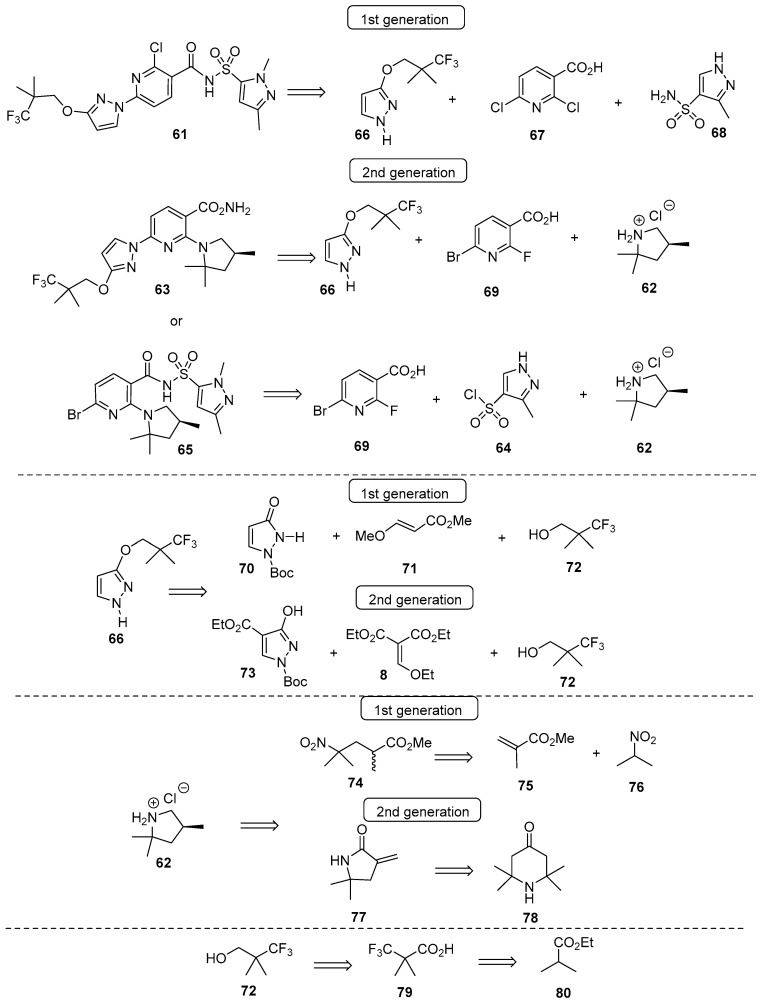
First and second generation for the synthesis of building blocks for elexacaftor (**3**).

Moreover, the synthesis of pyrazole **66** exemplified notable versatility, offering two distinct pathways for its formation. In the first generation, it originated through the connection of pyrazole **70** with methyl (*E*)-3-methoxyprop-2-enoate **71** and building block **72**. Alternatively, in the second generation, it resulted from the connection of pyrazole **73** with diethyl (2-ethoxymethylene)malonate **8** and building block **72**, requiring minimal adjustments. In addition, Vertex’s patent applications outlined two pathways to chiral pyrrolidine **62**. The first one involved an enzymatic hydrolytic resolution of the racemate **74**, prepared through the Michael addition of **76** to **75**. Alternatively, asymmetric hydrogenation was employed to introduce the asymmetric center of **62**, which was derived from piperidone **77**, previously prepared from **78**. Ultimately, intermediate **72** was obtained through the reduction of **79**, achieved via continuous flow photocatalyzed trifluoromethylation from **80** (Scheme 13).

**Scheme 14:**
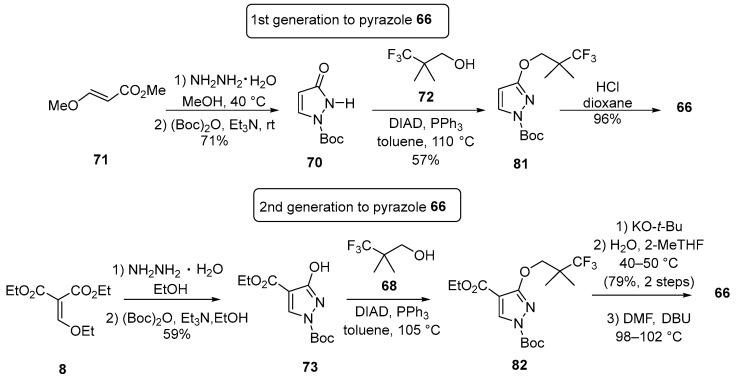
The first and second generations for obtaining pyrazole **66**.

The formation of pyrazole fragment **66** started with the combination of methyl (*E*)-3-methoxyprop-2-enoate **71** and hydrazine hydrate at 40 °C in MeOH for 1 h. The resulting mixture was then cooled to 20 °C, followed by the addition of triethylamine and (Boc)_2_O. After an aqueous workup, pyrazolone **70** was isolated through crystallization from heptane, yielding 71%. The Mitsunobu reaction between alcohol **72** and pyrazolone **70** in toluene at reflux led to pyrazole **81.** The presence of the bulky Boc group hinders *N*-alkylation, making it less favorable. The elevated temperature required for the reaction is attributed to the steric hindrance of neopentyl alcohol **72**, leading to a slow S_N_2 displacement reaction. Following the complete reaction, the solvent was changed to heptane to precipitate triphenylphosphine oxide, which was then filtered out. The purified product was obtained as a solid through flash chromatography with a 57% yield. The Boc group was eliminated by treating it with HCl in 1,4-dioxane, resulting in a 96% yield of pyrazole **66**. Due to the modest yield and purification challenges in the Mitsunobu reaction, an alternative approach to pyrazole **66** was explored. In this second method, diethyl (2-ethoxymethylene)malonate **8** reacted with hydrazine hydrate to form pyrazole **73**. Subsequent reactions with Boc_2_O and Et_3_N generated compound **82** with a 59% yield over two steps. The Mitsunobu reaction was conducted in toluene at 105 °C to produce **82**, which was not isolated but directly saponified with KO-*t*-Bu in 2-MeTHF/water. During the workup, the carboxylic acid derived from **82** was extracted into the aqueous layer, allowing for the convenient removal of Mitsunobu byproducts into the organic layer. Acidification and crystallization yielded a product with a 79% yield over two steps. Decarboxylation was achieved using 1,8-diazabicyclo[5.4.0]undec-7-ene (DBU) in DMF at 98−102 °C to obtain pyrazole **66**, with no specified yield provided for this step. Although this alternative route to pyrazole **66** in the second generation involves two additional steps starting from diethyl (2-ethoxymethylene)malonate **8**, it offers potential scalability advantages. The scalability is attributed to the ability to eliminate Mitsunobu byproducts (in Step 2) through extractive workup, thereby enhancing the overall feasibility of the synthetic process (Scheme 14).

**Scheme 15:**
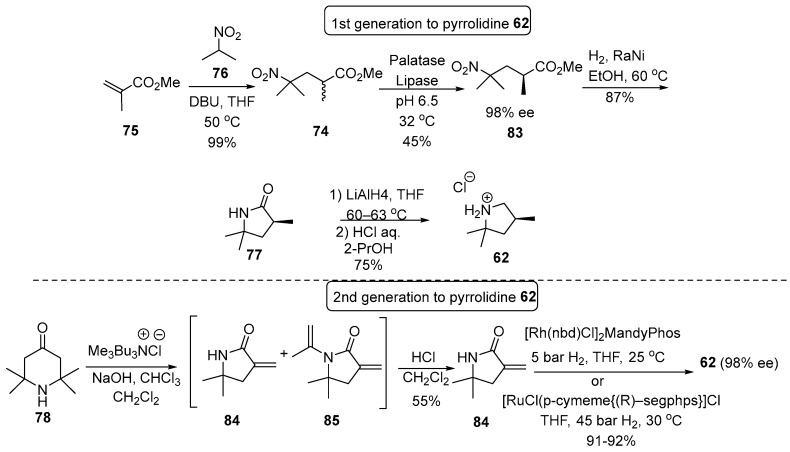
The first and second generations for obtaining pyrrolidine **62**.

For chiral pyrrolidine **62**, two routes were described in Vertex’s patent applications [110] as depicted in Scheme 15. The initial approach involved an enzymatic hydrolytic resolution. Initially, methyl methacrylate **75** was introduced to a THF solution containing 2-nitropropane **76** and DBU at 50 °C, resulting in the formation of Michael adduct **74** as a crude oil with a 99% yield after aqueous workup. Subsequently, Palatase lipase was employed for the enzymatic resolution of **74**, selectively hydrolyzing the undesired (*R*)-ester and leaving the desired (*S*)-ester **83** with a 98% enantiomeric excess. The (*R*)-acid was eliminated through aqueous extractive workup, and **83** was obtained as a crude oil with a 45% yield (90% of the available enantiomer). Reduction of the nitro group, coupled with cyclization to form lactam **77**, was achieved using Raney nickel in EtOH, resulting in an 87% yield after crystallization from heptane. Further reduction of the lactam to yield pyrrolidine **62** was carried out with LiAlH_4_ in THF, followed by crystallization of the HCl salt from 2-PrOH/MTBE, resulting in a 75% isolated yield. The overall yield for the four-step process amounted to 29%.

An alternate three-step route to **62** was described that employed an asymmetric hydrogenation to install the asymmetric center [110,111]. In the initial step, piperidone **78**, the phase-transfer catalyst (3.8%), chloroform (1.75 equiv), and dichloromethane (2 volumes) were blended at room temperature. Over 40 h, 50% aqueous NaOH was gradually added while maintaining reaction temperature between 15 and 25 °C to convert **78** into **84** and **85 [112]**. Following aqueous workup, the organic layer underwent treatment with 3N HCl to transform **85** into **84**. After solvent transition to 2-PrOAc, lactam **84** was obtained as a crystalline solid with a 55% yield. The Vertex patent applications feature an example conducted on a 257 kg scale of piperidone **78**. The asymmetric reduction of **84** was achieved using a Ru or a Rh catalyst at extremely low catalyst loadings. The Rh-catalyzed reduction was documented on a 6 kg scale of **84**, utilizing Mandyphos as the ligand (0.0046%) and chloronorbornadiene rhodium dimer (0.0015%) in THF with 5 bar H2 at 25 °C for 2 h.

Following the workup, product **62** was isolated through crystallization from heptane, yielding 91% with a 98% ee. The provided data did not specify the enantiomeric excess at the end of the reaction, making it unclear whether a significant ee enhancement occurred during crystallization. The example with the Ru catalyst was executed in a plug flow reactor on a 300 g scale. RuCl(p-cymene){(*R*)-segphos}]-Cl (0.02%) in THF and **84** in THF were introduced into the reactor at 30 °C with a residence time of 4 h and an H2 pressure of 45 bar. The reduction of lactam **84**, generated through asymmetric hydrogenation, was conducted with LiAlH4 in THF under similar reaction conditions as described earlier. Isolation of the HCl salt of pyrrolidine **62** was achieved through crystallization from either 2-PrOAc or 2-PrOAc/MTBE, yielding overall yields ranging from 78 to 89% with enantioselectivities ranging from 99.0 to 99.5% ee (Scheme 15).

**Scheme 16:**
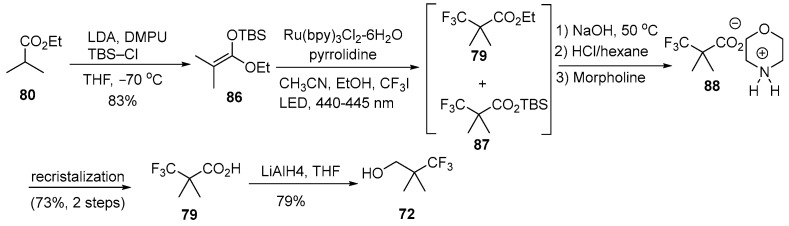
Preparation of alcohol **72**.

A novel continuous flow photocatalyzed trifluoromethylation method for synthesizing carboxylic acid **79** was outlined (Scheme 16). The process began with the deprotonation and silylation of ethyl isobutyrate **80**, resulting in the formation of silyl ketene acetal **86**. Trifluoromethylation was subsequently conducted in CH_3_CN/EtOH using Ru(bpy)_3_Cl_2_·6H_2_O (0.09 mol %) and a 440–445 nm light-emitting diode (LED) within a 52 mL photoreactor. After a 3 h continuous flow, a solution of 2.3 L was collected, representing slightly over 1 mol of products **79** and **87**. These products were then subjected to hydrolysis with NaOH, followed by the crystallization of **88** as its morpholine salt, resulting in a 73% isolated yield over the two steps [110,111].

**Scheme 17:**
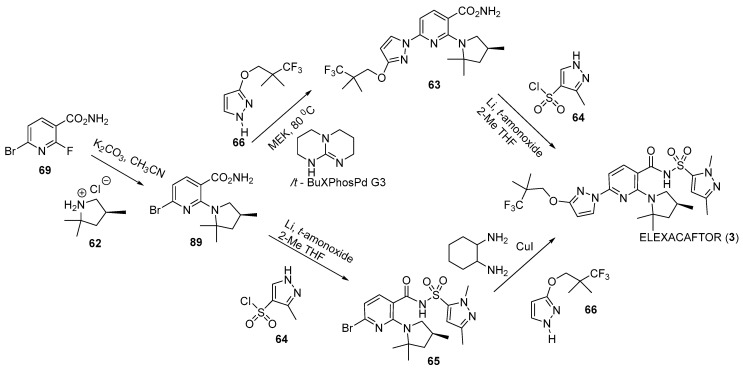
Two ways to obtain elexacaftor in the second generation (**3**).

In the first-generation route of elexacaftor (**3**), the yield reached approximately 29% over a linear sequence of seven steps, employing pivotal building blocks **66**, **67**, and **68** described in Scheme 13 [110,113]. A more streamlined and efficient strategy was elucidated in Vertex patent applications, starting with bromo-6-fluoronicotinic acid **69**. This compound underwent strategic transformation into nicotinamide **89** via an S_N_Ar reaction, displacing fluoride with pyrrolidine **62**. Subsequently, the formation of sulfonamide was initiated using sulfonyl chloride **64** and lithium *tert*-amoxide in 2-MeTHF, resulting in the production of **65**. This was followed by a C-N cross-coupling with pyrazole **66**, catalyzed by CuI and 1,2-diaminocyclohexane, leading to the synthesis of elexacaftor. Alternatively, the order of these two steps could be reversed. In this scenario, the C-N cross-coupling was facilitated by a Buchwald third-generation palladacycle (*t*-BuXPhosPd G3) to generate **63**, followed by sulfonamide formation, ultimately affording elexacaftor (**3**). Unfortunately, no specific yields were provided for any of these individual steps (Scheme 17). A summary of the synthetic methods and reaction conditions for the building blocks as well as elexacaftor are described in Supplementary Material (Supplementary Table S3).

### 4.2. Mechanisms of Action

Due to the modest clinical benefits of double combinations of CFTR modulators with a corrector (lumacaftor or tezacaftor) plus the potentiator ivacaftor, additional HT screenings were performed to identify next-generation correctors to further increase the rescue of CFTR in triple combinatorial treatments. Veit and colleagues [114] identified three small-molecule series acting on NBD1, NBD2, and the corresponding TMD interfaces by performing HT screenings. The authors isolated and characterized novel p.Phe508del-CFTR correctors with three distinct MoAs, assessing the effects of combinations with these compounds. Although individually the correctors only exhibited limited rescue of the p.Phe508del variant, synergistic effects were observed for the combination of these compounds targeting different structural defects on the mutant protein [114].

Efforts on the CF drug development pipeline swiftly led to the identification of elexacaftor and the clinical approval in 2019 by the FDA and in 2020 by the EMA of the triple combinatorial treatment ivacaftor/tezacaftor/elexacaftor—the most effective therapy in rescuing mutant CFTR nowadays. This combination was initially approved for PwCF carrying at least one p.Phe508del allele; however, theratyping efforts (i.e., matching modulators to responsive CFTR variants) led to label extension approval by the FDA to >170 CF-causing variants based on in vitro data [20,115].

To explore the MoA of elexacaftor, Veit and co-authors [51] assessed this corrector in combination with other compounds in human bronchial epithelial cells. It was observed that elexacaftor had synergistic effects with type I (NBD1:TMD1/2 interfaces) and II (NBD2 and/or its interfaces) correctors in rescuing the processing defect of p.Phe508del and rare folding variants. These findings suggested a direct pharmacochaperone activity for elexacaftor as a type III CFTR corrector and an allosteric correction mechanism for the triple combination [51]. In a subsequent study, Veit and colleagues [116] further explored the rescue effects of elexacaftor in combination with tezacaftor on three rare CFTR folding variants: p.Pro67Leu-, p.Leu206Trp- and p.Ser549Arg-CFTR (legacy names *P67L*, *L206W*, and *S549R*, respectively). Whereas this double corrector treatment significantly increased the correction efficacy of the first two variants, it only showed modest effects on the third, which were comparable to tezacaftor alone. These findings suggested that not all PwCF carrying rare CFTR missense variants may have an additional clinical benefit with triple combination treatment compared to the double one.

Capurro and co-authors [117] further explored the efficacy and properties of elexacaftor in rescuing p.Phe508del in combination with type I (lumacaftor, tezacaftor) and type II (corr-4a, 3151) correctors. It was observed that this modulator had additive effects with the other four correctors, providing additional evidence for a type III correction mechanism for elexacaftor. Despite the highly significant rescue of p.Phe508del, the authors observed that none of the evaluated combinations of correctors fully prevented the folding, trafficking, or stability defects of this variant [117]. Additionally, Becq and collaborators [118] investigated the effects of correctors lumacaftor, tezacaftor, or elexacaftor individually and the combination of tezacaftor and elexacaftor with or without the potentiator ivacaftor by performing biochemical and functional assays in p.Phe508del-expressing airway epithelial cells. The authors confirmed that p.Phe508del maturation and trafficking correction by double corrector treatment was reduced in the presence of ivacaftor, as previously reported [51,117]. This evidence suggested that triple combination with a different potentiator may elicit further correction effects and improve clinical effectiveness [118].

Recent studies investigating the MoA of elexacaftor have shown that in addition to corrector activity, it also acts as a weak CFTR potentiator [68,119,120]. Laselva and colleagues [119] first described the dual activity of elexacaftor as both corrector and potentiator in HEK293 and primary nasal epithelial cells from PwCF homozygous for p.Phe508del, p.Gly85Glu, p.Met1101Lys, or p.Asn1303Lys (legacy names *F508del*, *G85E*, *M1101K*, or *N1303K*). Potentiator activity of elexacaftor was different for the distinct variants but it was significantly less effective than that of ivacaftor for p.Phe508del [119]. The authors suggested that depending on the genotype, rescue by elexacaftor could be due to one or both of its modulator activities. Concurrently, Veit and co-authors [68] also explored the putative potentiator activity of elexacaftor, investigating its effects individually and in combination with ivacaftor in p.Phe508del and class III gating variants p.Gly551Asp and p.Gly1244Glu (legacy names *G551D* and *G1244E*). They observed that acute addition of both elexacaftor and ivacaftor elicited higher current levels of p.Phe508del- and p.Gly551Asp-CFTR in primary human bronchial and nasal epithelia. Additionally, combinatorial profiling was performed and suggested a distinct MoA for elexacaftor due to its (at least) additive effects with ivacaftor [68]. Nevertheless, Tomati and collaborators [121] also investigated the rescue of p.Gly1244Glu by the approved triple therapy, as the gating defect caused by this variant is not completely rescued by ivacaftor. The authors performed molecular, biochemical, and functional analyses, and the results evidenced dependence on the cell background for the rescue of p.Gly1244Glu. In heterologous expression systems, elexacaftor acted as a co-potentiator, whereas in primary nasal epithelial cells, the modulator did not exert that potentiator activity but rather increased p.Gly1244Glu-CFTR processing, suggesting a combined processing/gating defect for this variant [121].

Shaughnessy and collaborators [120] further investigated and characterized the acute and chronic treatment, pharmacology properties, and efficacy of CFTR potentiation by elexacaftor in WT and classes II, III, and IV CFTR variants. They observed that this modulator exhibited an effective potentiator activity both in normal and mutant CFTR. Furthermore, the authors showed that the potentiation activity of elexacaftor had multiplicative synergistic effects with ivacaftor in mutant CFTR, suggesting different MoA’s for the potentiation action of these two modulators [120]. In a subsequent study by the same authors [122], the effects of the combination of tezacaftor and elexacaftor with or without prolonged ivacaftor exposure (24 h) were assessed in p.Phe508del-expressing human nasal epithelial cells by Ussing chamber measurements. Constitutive CFTR-mediated ion transport was only observed to increase in cells treated with the double corrector combination and prolonged co-treatment with ivacaftor. These findings indicated that this triple combination therapy increases constitutive CFTR activity, leading to a net increase in CFTR function, possibly explaining the high clinical effectiveness of this treatment [122].

**Figure 5:**
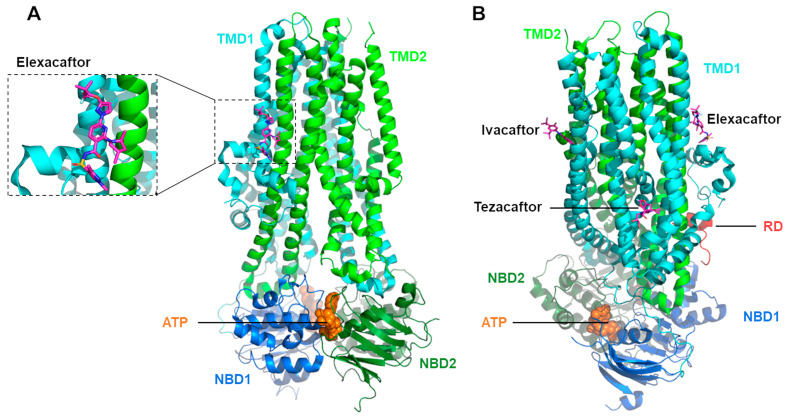
**Molecular structure of phosphorylated, ATP-bound human CFTR in complex with CFTR modulators.** Ribbon diagrams of the complex of human CFTR with (**A**) elexacaftor and (**B**) the triple combination of elexacaftor, tezacaftor, and ivacaftor. Adapted from PDB: 8EIG and PDB: 8EIQ [123], respectively, using PyMol Version 2.5.7.

As the mechanism of action of elexacaftor remained not fully understood, Baatallah and colleagues [99] explored possible binding site(s) for this modulator. The authors found two potential binding sites for elexacaftor located on TMD1 and NBD1. Although the first site on TMD1 was uniquely identified to elexacaftor, the second on NBD1 was demonstrated to be a preferential binding site shared with lumacaftor (previously mentioned in Section 3.2) [99]. Because the double combination of elexacaftor and lumacaftor elicited high levels of CFTR correction in all assessed variants, the authors thus suggested that binding of these correctors to distinct sites on TMD1 enhanced the allosteric coupling between the CFTR domains, TMD1 and NBD1, resulting in the increased efficacy observed for the double corrector combination [99]. Similarly to what was described for lumacaftor and tezacaftor, Fiedorczuk and Chen [123] determined the cryo-EM structures of human CFTR in complex with elexacaftor alone (PDB: 8EIG; Figure 5A), with both elexacaftor and lumacaftor (PDB: 8EIO), and with the triple combination of elexacaftor, tezacaftor, and ivacaftor (PDB: 8EIQ; Figure 5B). These molecular structures provide further insights into the MoA of these modulators, revealing the synergistic rescue of p.Phe508del-CFTR by triple modulator treatment, whose compounds bind to distinct sites on this mutant protein [123]. In combination with lumacaftor or tezacaftor, elexacaftor was found to rectify interdomain assembly defects in p.Phe508del and to stabilize the NBD1:TMD interface [123]. The authors determined the binding site of elexacaftor, comprising residues in TMD1 (lasso motif) and TMD2 (Helices 10 and 11), and resulting in partial stabilization of NBD1. At the same time, Wang and co-authors [124] used cryo-EM global conformational ensemble reconstruction to investigate the dual action of elexacaftor as a corrector and a potentiator. A binding site mediating correction and potentiation was observed for this CFTR modulator in a cavity formed by the lasso motif and transmembrane Helices 2, 10, and 11 [124]. Binding to this site by elexacaftor not only mediates its corrector activity, but also explains the potentiator effect due to the shift towards open-channel conformations [124].

Concurrently, Hillenaar and collaborators [125] performed biosynthetic radiolabeling combined with protease susceptibility assays to provide further evidence on the CFTR domain(s) involved in elexacaftor-promoted modulation. The stability of full-length protein, the individual domains, and truncated p.Phe508del-CFTR constructs was evaluated. For individual CFTR domains, elexacaftor did not lead to higher intracellular stability, whereas in p.Phe508del-CFTR the modulator elicited stability improvement [125]. These findings suggested that elexacaftor most likely requires interaction with more than a single domain to exert its rescue effects, in an MoA probably affecting domain interfaces such as TMD1 and TMD2 assembly and their interface with NBD1 [125]. Bongiorno and co-authors [126] further explored the MoA of elexacaftor by assessing its effects on p.Phe508del-CFTR to identify the protein domains affected by this modulator. Using different biochemical approaches and plasmid constructs expressing the isolated domains, the authors suggested that different correctors can act on the expression and stability of distinct p.Phe508del-CFTR regions, identifying TMD2 as the primarily CFTR domain involved in p.Phe508del rescue by elexacaftor [126].

The MoA of novel compounds can be assessed by investigating their possible additive effects to previously characterized CFTR genetic revertants. While validating p.Phe508del rescue by novel correctors among triazole compounds, Bacalhau and colleagues [127] evaluated elexacaftor as a positive control for this rescue. The authors found that elexacaftor was additive to p.Gly550Glu, p.Arg1070Trp and 4RK in rescuing p.Phe508del processing and function, thus evidencing a distinct MoA for this modulator, not acting on the NBD1:NBD2 interface or the NBD1:ICL4 interface nor the arginine frame tripeptide-dependent ER retention mechanisms, respectively [127].

Stanke and co-authors [128] investigated the effects on CFTR protein expression by the approved triple therapy in rectal biopsies from 21 PwCF p.Phe508del homozygous or compound heterozygous before and during treatment. It was observed that this therapy increased CFTR protein expression and maturation in the majority of samples from PwCF evaluated, which suggested that this combination facilitates the posttranslational processing of some mutant CFTR, although not to the fully-glycosylated levels of WT-CFTR [128].

Although CFTR modulator treatment is approved for the majority of PwCF, there are still individuals without causal treatments or with limited clinical benefits from approved therapies. Ensinck and co-authors [70] explored combinations of approved and investigational CFTR modulators for the rescue of variants p.Gly85Glu and p.Asn1303Lys in primary rectal organoids using the forskolin-induced swelling (FIS) assay. Novel combinations were found to increase the rescue of these variants beyond the approved triple modulator therapy, with maximal efficacy observed for p.Gly85Glu by elexacaftor and corr-4a combination and for p.Asn1303Lys by the quadruple combination of tezacaftor, elexacaftor, ivacaftor, and apigenin [70]. These findings suggested that elexacaftor acts on these variants by distinct MoAs, rescuing CFTR folding and function in p.Gly85Glu and improving the gating defect of p.Asn1303Lys in the presence of ivacaftor [70]. Concurrently, McKee and co-authors [129] compared the effects of elexacaftor and tezacaftor individually and in a double combination on the PM expression of 129 CF-causing variants using a deep mutational scanning approach to compare mutation-specific effects by these CFTR modulators. In HEK293T cells, the authors observed that, in the presence of elexacaftor or tezacaftor individually, these modulators are most effective in variants with defects near their respective binding sites and with intermediate PM expression [129]. Additionally, the double corrector combination was observed to synergistically enhance the PM expression of several variants, heterogeneously expressed and distributed throughout the CFTR structure [129]. Furthermore, Kim and collaborators [130] investigated the effects of elexacaftor in variants p.Pro67Leu (lasso motif) and p.Leu206Trp (transmembrane Helix 3). It was observed that both variants were hyper-responsive to lumacaftor, whereas p.Pro67Leu was only minimally rescued by elexacaftor [130]. Additionally, the authors measured proteostasis changes to quantify the interactomics of these variants in response to elexacaftor. Interestingly, this modulator was found to decrease early CFTR biogenesis interactions (translation, folding, and degradation) for p.Leu206Trp but without impact on p.Pro67Leu, suggesting that elexacaftor exerts its function on CFTR after early quality control checkpoints, namely translation, and folding [130]. In parallel, Lefferts and colleagues [131] assessed the efficacy of triple modulator therapy in 22 patient-derived intestinal organoids with rare CFTR variants at the time not eligible for modulator treatment. Using the FIS assay to measure CFTR-dependent fluid secretion, it was found that triple therapy rescued CFTR function in 12 organoids with 11 unique CF genotypes, identifying additional rare variants with potential clinical benefit for this modulator therapy.

Recently, Im and collaborators [132] investigated TMD folding and assembly using CFTR as a model protein. After performing kinetic radiolabeling and protease susceptibility assays, it was found that the CFTR folding process involves two different steps, the first co-translational and the second post-translational, and a global folding profile of this protein was reported. In addition, the authors assessed the influence of lumacaftor and elexacaftor on the p.Phe508del-CFTR folding pathway, observing that elexacaftor increased CFTR transport to the Golgi, while the double corrector combination boosted domain assembly, although without fully correcting NBD1 folding [132]. Soya and co-authors [133] further explored the molecular and cellular mechanisms involved in the folding landscape biogenesis of ABCC sub-family transporters and the mechanism of elexacaftor-mediated CFTR rescue. Using molecular dynamic simulations and biochemical and HDX assays, they found that stabilization of NBD1:TMD1/2 interdomain coupling leads to higher CFTR post-translational cooperative domain folding [133]. The authors proposed that dynamic allosteric domain–domain coupling modulates ABCC transporters’ folding landscape and suggested a two-step simplified folding model for ABCC transporters [133]. More recently, Ersoy and colleagues [65] investigated long-range allosteric communications in CFTR by computational analysis integrating the Gaussian network model, transfer entropy, and anisotropic normal mode Langevin dynamics. The authors used the previously published structure of p.Phe508del-CFTR bound to ivacaftor, tezacaftor, and elexacaftor [123] to explore the allosteric effects of these modulators. They observed that while tezacaftor binds to residues that are not allosteric sources (termed “valleys”), the binding sites for ivacaftor and elexacaftor comprise some residues that act as main allosteric sources, suggesting a role for these compounds as allosteric modulators [65].

While this manuscript was under review, a new study was published and demonstrated that PTI-801 (posenacaftor)—a third-generation corrector—shares a common mechanism with elexacaftor to rescue p.Phe508del-CFTR [134]. By employing biochemical, fluorescence microscopy, and functional assays, PTI-801 has been demonstrated to rescue p.Phe508del-CFTR with greater correction effects in combination with type I correctors (tezacaftor, lumacaftor, ABBV-2222, or FDL-169), but not with elexacaftor. PTI-801 and elexacaftor also displayed similar behavior on genetic revertants of p.Phe508del-CFTR (p.Val510Asp, p.GlyG550Glu, p.Arg1070Trp, and 4RK), and their rescue effects were abrogated in the *in cis* p.Arl1102Ala [134]. These findings suggest that PTI-801 can be a feasible alternative for the development of novel modulator combinations, aiming to attain greater rescue of CFTR variants. 

## 5. Concluding Remarks

This comprehensive review describes the retrosynthetic analysis as a key aspect of the synthesis of ivacaftor, tezacaftor, and elexacaftor, with a specific focus on the most recent synthetic routes pioneered by Vertex Pharmaceuticals. Regarding ivacaftor, in addition to Vertex’s established route, several other companies have explored diverse synthetic pathways to acquire the essential quinoline building block. Simultaneously, various research groups have dedicated their efforts to refining the synthesis of the aminophenol and quinoline moieties. Tezacaftor, possessing a more intricate structure compared to ivacaftor, sees its second generation as the presumed commercially viable option. The synthetic route for tezacaftor entails 15 steps, arranged in a linear sequence of 7 steps. Elexacaftor, surpassing ivacaftor in complexity, boasts two described synthetic routes, although details regarding the commercial route remain undisclosed. In one generation, a yield of approximately 29% was achieved over a linear sequence of seven steps. Vertex’s patent applications articulate a more streamlined and efficient strategy, commencing with bromo-6-fluoronicotinic acid **54** and employing pyrazole **52** and amine **51**. Regrettably, specific yields for individual steps within this advanced strategy remain undisclosed.

The discovery and clinical approval of molecules acting as CFTR modulators have led to a new era in CF management and treatment over the last decade. In particular, the groundbreaking triple combinatorial therapy has shown remarkable improvements in lung function and overall clinical features, thus significantly enhancing the quality of life for the majority of PwCF. Several clinical studies have been performed to demonstrate the safety and efficacy/effectiveness of the CFTR modulator combinations as well as evaluate the corresponding benefits and adverse effects. Another review [135] extensively elaborates and discusses the major therapeutic benefits and adverse effects reported to date in the treatment of PwCF with these approved modulator therapies.

Additionally, various studies have focused on shedding light on the molecular mechanisms underlying the action of these CFTR modulators. Despite the advances in the understanding of their MoA, several mechanistic gaps remain. As not all PwCF are eligible for the approved therapies, or even if eligible, do not currently receive them due to the associated high costs [19,136,137], understanding the MoA of these modulators is paramount for the development of novel therapeutic strategies. Comprehension of the MoA of CFTR modulators provides insights into how they interact with mutant CFTR and the specific defects these modulators address, thus opening new avenues for the identification of potential drug targets within the CFTR structure. As the CF treatment landscape continues to evolve, consolidating the knowledge of the synthetic routes and MoA of CFTR modulators may provide valuable insights for the development of alternate, more efficacious, and/or cost-effective CF therapies, expanding the range of variants amenable to modulation.

## Supplementary Materials

The following supporting information can be downloaded at: https://www.mdpi.com/article/10.3390/molecules29040821/s1. Supplementary Table S1: Summary of the synthetic methods and conditions of the reactions of ivacaftor; Supplementary Table S2: Summary of the synthetic methods and conditions of the reactions of tezacaftor; Supplementary Table S3: Summary of the synthetic methods and conditions of the reactions of elexacaftor.

## Abbreviations

ABC—ATP-binding cassette; ABCC—ABC sub-family C; ATP—adenosine triphosphate; CF—cystic fibrosis; CFTR—CF transmembrane conductance regulator; cryo-EM—cryogenic electron microscopy; DBU—1,8-diazabicyclo[5.4.0]undec-7-ene; DMAP—catalytic 4-(dimethylamino)pyridine; DMF—dimethylformamide; EMA—European Medicines Agency; FDA—Food and Drug Administration; FIS—forskolin-induced swelling; FRET—fluorescence resonance energy transfer; HATU—hexafluorophosphate; HDX—hydrogen/deuterium exchange; HT—high-throughput; ICL—intracellular loop; LED—light-emitting diode; MoA—mechanism of action; MTBE—methyl tert-butyl ether; NBD—nucleotide-binding domain; PKA—protein kinase A; PM—plasma membrane; Po—open probability; PPA— polyphosphoric acid; PwCF—people with CF; RD—regulatory domain; SAR—structure-activity relationship; T3P—propanephosphonic acid anhydride; TMD—transmembrane domain; WT—wild type.
