# Supplementary material for: Organic Synthesis and Current Understanding of the Mechanisms of CFTR Modulator Drugs Ivacaftor, Tezacaftor, and Elexacaftor
Source: Molecules. 2024 Feb 10;29(4):821. doi: 10.3390/molecules29040821 (PMC10891718; doi:10.3390/molecules29040821)
Supplement: Supplementary file 1 [file molecules-29-00821-s001.zip › molecules-2827653-supplementary.pdf]

Supplementary Material

Organic Synthesis and Current Understanding of the Mechanisms of the CFTR Modulator Drugs Ivacaftor, Tezacaftor, and Elexacaftor

Filipa C. Ferreira [1], Camilla D. Buarque [2,*] and Miquéias Lopes-Pacheco [1,*]

[1] Biosystems & Integrative Sciences Institute (BioISI), Faculty of Sciences University of Lisbon, 1749-016 Lisbon, Portugal
[2] Department of Chemistry, Pontifical Catholic University of Rio de Janeiro (PUC-Rio), 22435-900 Rio de Janeiro, RJ, Brazil
* mlpacheco@fc.ul.pt (M.L.-P.), camilla-buarque@puc-rio.br (C.D.B.)

Supplementary Table S1. Summary of the synthetic methods and conditions of the reactions of ivacaftor

Approach	Substrate A	Substrate B	Reaction conditions	Product
1	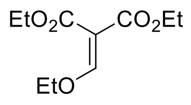 **8**	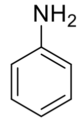 **9**	1) 140-150 °C, 2h<br>2) $POCl_3$, PPA, 70 °C, 4h (70%)<br>3) NaOH, reflux, 92%	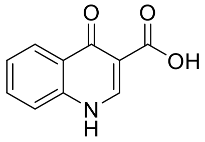 **4a**
2	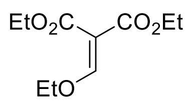 **8**	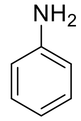 **9**	1) 110 °C, 2.5h, neat<br>2) phenyl ether 228-232 °C, 1.5h<br>3) 2N NaOH, ethanol, reflux, 16h	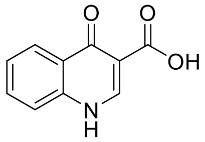 **4a**
1	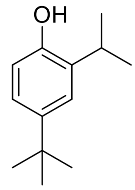 **21**	–	1) 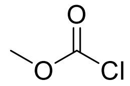 Et_3N, DMAP, CH_2Cl_2, 0-25 °C (100%)<br>2) HNO_3/H_2SO_4<br>3) KOH/MeOH (2 steps) (29%)<br>4) HCO_2NH_4 EtOH, Pd/C (100%)	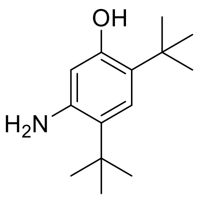 **5a**
2	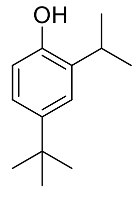 **21**	–	1) 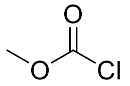 Et_3N, DMAP, CH_2Cl_2, 0-25 °C (100%)<br>2) HNO_3/H_2SO_4,CH_2Cl_2,-5-0 °C<br>3) Pd/C, H_2, MeOH 25 °C	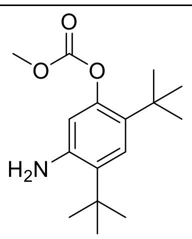 **5b**
1	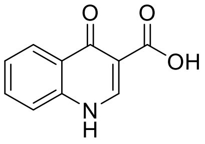 **4a**	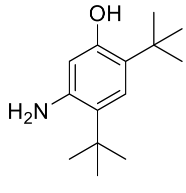 **5a**	HATU, Et_3N, DMF (71%)	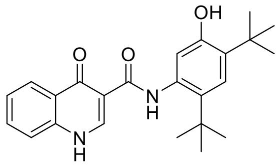 **IVACAFTOR (1)**
2	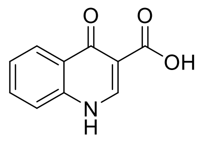 **4a**	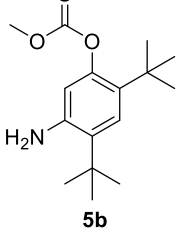 **5b**	1) T3P, pyr, 2-MeTHF<br>2) NaOH, MeOH	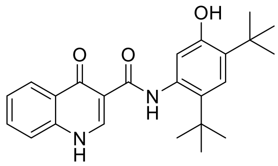 **IVACAFTOR (1)**

Supplementary Table S2. Summary of the synthetic methods and conditions of the reactions of tezacaftor

Approach	Substrate A	Substrate B	Reaction conditions	Product
1	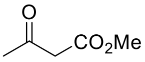 **36**	–	1) NaH, MeI, THF (53%)<br>2) PCl5, DMF (cat), CH_2Cl_2, reflux (82%)<br>3) aq. NaOH, reflux (44%)<br>4) $NaNH_2$, DMSO (94%)<br>5) BnOH, DCC, CH_2Cl_2 (59%)	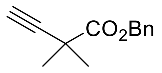 **30**
1	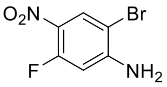 **28**	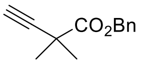 **30**	1) $Pd(PPh_3)_2Cl_2$, Et_3N, CuI, 80 °C (56%)<br>2) $PdCl_2$, CH_3CN, 80 °C (90%)<br>3) 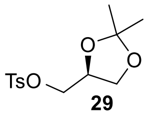 **29**, Cs_2CO_3, DMF, 80 °C<br>4) $LiAlH_4$, THF (87%, 2 steps)<br>5) H_2, Pd/C, EtOH (78%)	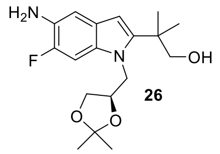
2	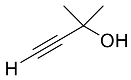 **47**	–	1) HCl conc. (90%)<br>2) a) Mg, THF; b) 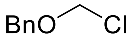<br>3) KOH, MeOH	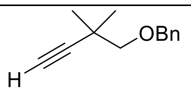 **33**
2	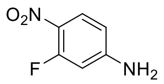 **31**	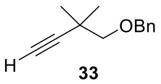 **33**	1) NBS, EtOAc (50%)<br>2) 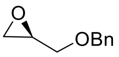, $Zn(ClO_4)_2.2H_2O$, toluene, 4Å MS, 80°C<br>3) a) H_2, Pt(S)/C, *i*-PrOAc, 30 °C; b) p-TsOH-H_2O, CH_2Cl_2<br>4) **33**, $Pd(OAc)_2$, dppb, K_2CO_3, CH_3CN, 80 °C	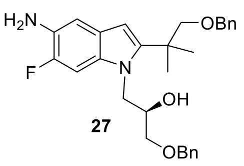
1	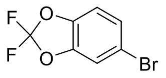 **34**	–	1) CO, Et_3N, $Pd(PPh_3)4$, CH_3CN, MeOH, 75 °C<br>2) $LiAlH_4$, THF (76%, 2 steps)<br>3) a) $SOCl_2$, CH_2Cl_2; b) NaCN, DMSO<br>4) 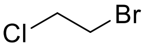, BnNEt3Cl, NaOH, 70 °C<br>5) 10% aq. NaOH, reflux (1.6%, 4 steps)	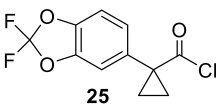 **25**
1	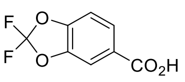 **35**	–	1) a) $NaAlH_2(OCH_2CH_2OCH_3)$, toluene; b) 10% aq. NaOH (86-92%)<br>2) $SOCl_2$, DMAP, MTBE (82-100%)<br>3) NaCN, DMSO, 30-40 °C (95-100%)<br>4) 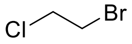,Oct_4NBr, 50% KOH, 70 °C (88-100%)<br>5) 6N NaOH, EtOH, 80 °C (69%)<br>6) $SOCl_2$, DMF	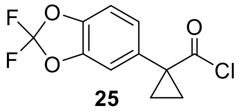 **25**

2	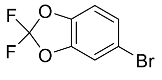<br>**34**	–	1) NC⌒CO$_2$Et ,Pd(dba)$_2$, *t*-Bu$_3$P, 70 ^{o}C, Na$_3$PO$_4$, tol/H$_2$O<br>2) HCl 3N, DMSO, 75 ^{o}C (66%, 2 steps)<br>3) Cl⌒Br ,Oct$_4$NBr, 50% KOH, 70 ^{o}C (88-100%)<br>4) 6N NaOH, EtOH, 80 ^{o}C (69%)<br>5) SOCl$_2$, DMF	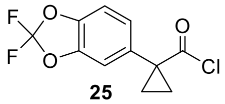<br>**25**
1	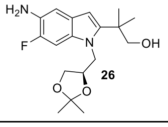	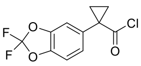<br>**25**	1) Et$_3$N, CH$_2$Cl$_2$ (100%)<br>2) *p*-TsOH-H$_2$O, MeOH, H$_2$O, 80 °C (47%)	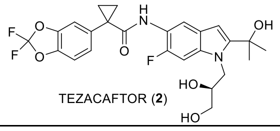
2	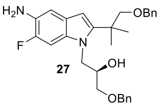	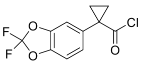<br>**25**	1) Et3N, toluene/CH$_2$Cl$_2$ (100%)<br>2) H2, Pd/C, MeOH (63-84%)	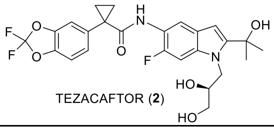

Supplementary Table S3. Summary of the synthetic methods and conditions of the reactions of elexacaftor

Approach	Substrate A	Substrate B	Substrate C	Reaction conditions	Product
1	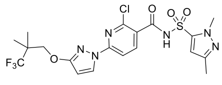 **61**	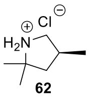 **62**	–	K_2CO_3, DMSO, DEE, 120 °C<br>90%	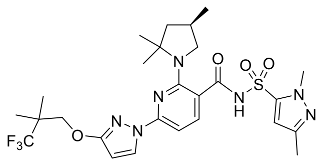 ELEXACAFTOR (**3**)
1	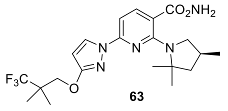 **63**	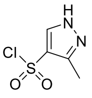 **64**	–	Li, *t*-amonoxide, 2-MeTHF	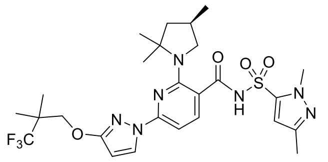 ELEXACAFTOR (**3**)
2	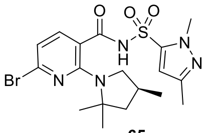 **65**	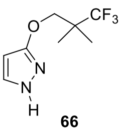 **66**	–	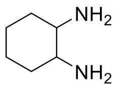 /CuI	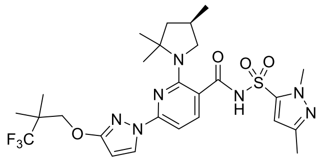 ELEXACAFTOR (**3**)
1	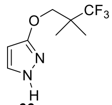 **66**	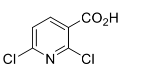 **67**	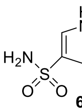 **68**	1) K_2CO_3, DABCO, DMF, rt (99%)<br>2) CDI, DBU, THF (93%)	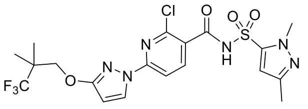 **61**
2	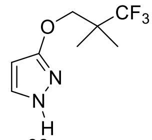 **66**	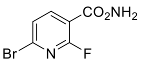 **69**	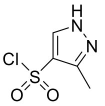 **64**	MEK, 80°C,<br>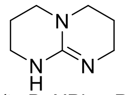<br>/*t* - BuXPhosPd G3	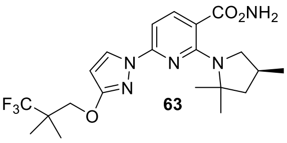 **63**
2	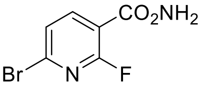 **69**	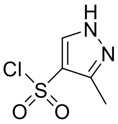 **64**	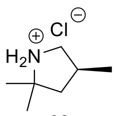 **62**	Li, *t*-amonoxide, 2-MeTHF	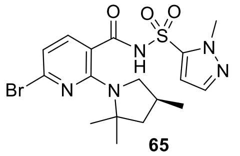 **65**
1	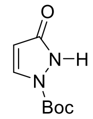 **70**	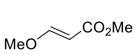 **71**	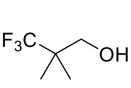 **72**	1) a) NH_2NH_2. H_2O, MeOH, 40 °C; b) 2) $(Boc)_2O$, Et3N, rt (71%)<br>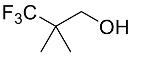 **72**<br>2)<br>3)DIAD, PPh_3, toluene, 110 °C (57%)	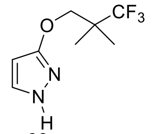 **66**

2	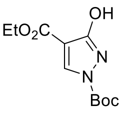<br>**73**	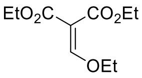<br>**8**	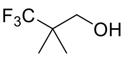<br>**72**	1)a) NH_2NH_2. H_2O, EtOH; b) 2) (Boc)2O, Et3N, rt (59%)<br>2)DIAD, PPh_3, toluene, 105 °C<br>3) a) KO-t-Bu<br>b) H_2O, 2-MeTHF, 40-50 °C, (79%, 2 steps)	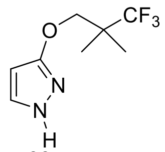<br>**66**
1	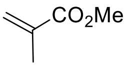<br>**75**	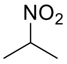<br>**76**	–	1) 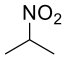 **76** DBU, THF, 50 °C (99%)<br>2) Palatase Lipase, pH 6.5, 32 °C (45%)<br>3) H_2, RaNi, EtOH, 60 °C (87%)<br>4) a) LiAlH4, THF, 60-63 °C; b) 2) HCl aq.2-PrOH (75%)	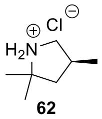<br>**62**
2	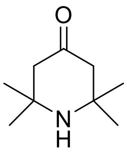<br>**78**	–	–	1) Me3Bu3NCl, NaOH, $CHCl_3$, CH_2Cl_2<br>2) HCl, CH2Cl_2 (55%)<br>3) [Rh(nbd)Cl]2MandyPhos, 5 bar H2, THF, 25 oC or [RuCl(p-cymeme{(R)-segphps}]Cl, THF, 45 bar H2, 30 °C (91-92%)<br>4) a) LiAlH4, THF, 60-63 oC; b) 2) HCl aq.2-PrOH (75%)	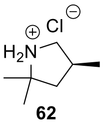<br>**62**
2	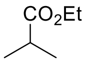<br>**80**	–	–	1) LDA, DMPU, TBS-Cl, THF, -70 oC (83%)<br>2) Ru(bpy)3Cl2-6H2O, pyrrolidine, CH3CN, EtOH, CF3I, LED, 440-445 nm<br>3) a) NaOH, 50 oC, b) HCl/hexane, 3) Morpholine (73%)<br>4) LiAlH4, THF (79%)	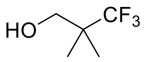<br>**72**
